# Determination and Imaging of Small Biomolecules and Ions Using Ruthenium(II) Complex-Based Chemosensors

**DOI:** 10.1007/s41061-022-00392-8

**Published:** 2022-06-13

**Authors:** Miaomiao Wu, Zexi Zhang, Jiaxi Yong, Peer M. Schenk, Dihua Tian, Zhi Ping Xu, Run Zhang

**Affiliations:** 1 Australian Institute for Bioengineering and Nanotechnology, The University of Queensland, Brisbane, QLD 4072 Australia; 2 School of Agriculture and Food Sciences, The University of Queensland, Brisbane, QLD 4072 Australia

**Keywords:** Ru(II) complexes, Chemosensors, Luminescent imaging, Ions, Small biomolecules detection

## Abstract

Luminescence chemosensors are one of the most useful tools for the determination and imaging of small biomolecules and ions in situ in real time. Based on the unique photo-physical/-chemical properties of ruthenium(II) (Ru(II)) complexes, the development of Ru(II) complex-based chemosensors has attracted increasing attention in recent years, and thus many Ru(II) complexes have been designed and synthesized for the detection of ions and small biomolecules in biological and environmental samples. In this work, we summarize the research advances in the development of Ru(II) complex-based chemosensors for the determination of ions and small biomolecules, including anions, metal ions, reactive biomolecules and amino acids, with a particular focus on binding/reaction-based chemosensors for the investigation of intracellular analytes’ evolution through luminescence analysis and imaging. The advances, challenges and future research directions in the development of Ru(II) complex-based chemosensors are also discussed.

## Introduction

Small biomolecules and ions are building blocks of living organisms and play indispensable roles in all biological processes, such as enzymatic reactions, metabolism, growth, adaptation, and various disease developments and progressions [1, 2]. Determination and monitoring of the levels of these biomolecules and ions in situ are essential for better understanding their biological roles in biomedical systems, thus contributing to early diagnosis and treatment assessment of various diseases [3–5]. The most common and typical methods, such as high-performance liquid chromatography (HPLC) and inductively coupled plasma-optical emission spectroscopy/mass spectrometry (ICP-OES/MS), have been developed for the determination of small biomolecules and ions in vitro [6–8]. Onsite determination of these analytes in situ and in vivo, using these techniques, is not possible because the sample preparation in solution is an essential step to ensure performing successful analysis [9]. Imaging technologies, such as computerized tomography (CT) [10], magnetic resonance imaging (MRI) [11] and positron emission tomography (PET), have been widely used in clinical diagnosis [12], while these technologies cannot be directly used for the determination of the concentration and/or activity of these analytes in biological samples [13, 14]. This is mainly because the contrast agents (CAs) used in these technologies are generally nonspecific; more importantly, these CAs hardly respond to small biomolecules and ions at molecular level because of their resolution and sensitivity limitations [15]. Other approaches to optical detection, such as fluorescence and phosphorescence measurements, have also been successfully developed and adopted in biomedical research and clinical diagnosis [16]. In contrast to conventional bioassay and imaging technologies, luminescence bioassay and imaging using advanced optical spectroscopic and imaging instruments are featured with high sensitivity and selectivity, fast response time and low cost, enabling their use in biological and biomedical investigations involving in vitro bioassay and in vivo luminescence bioimaging [17–20].

**Fig. 1:**
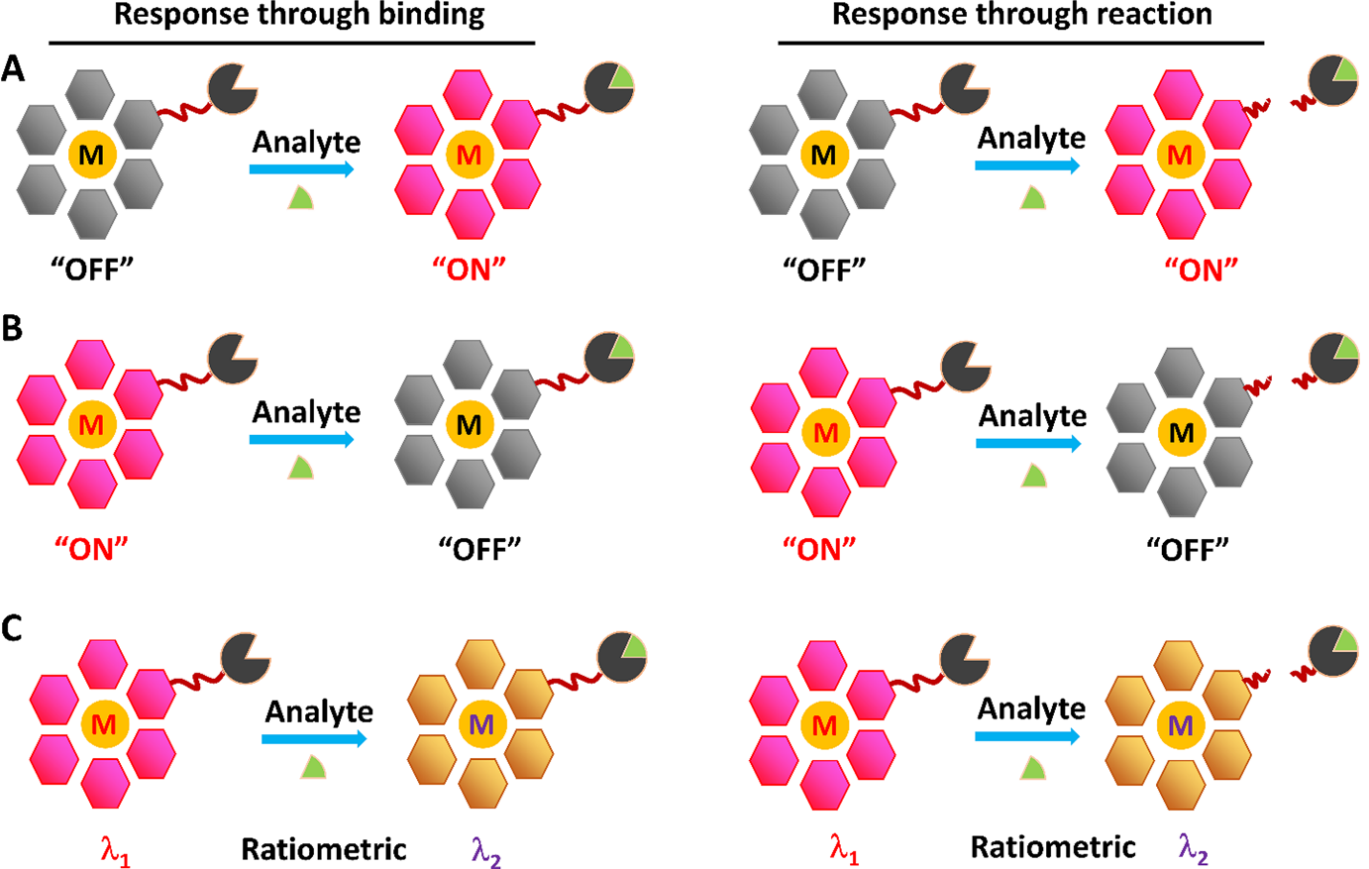
Design of chemosensors for the determination of analytes through the response mechanisms of binding and reaction, resulting in “OFF–ON” (**A**), “ON–OFF” (**B**), and ratiometric (**C**) luminescence response to analytes

Chemosensors are one of the most important tools for luminescence bioassay and imaging of small biomolecules and ions in situ in real time [21–23]. Luminescent chemosensors are normally designed as chemical compounds that can respond to targeted analytes through a unique binding/reaction (Fig. 1) [24, 25]. Generally, the chemosensors with reaction-based sensing mechanisms have higher selectivity, and the chemosensors with binding-based sensing mechanisms feature excellent reversibility for monitoring the targeted analyte in situ. As a result of these response processes, the luminescence signals can be switched “ON” (Fig. 1A) or “OFF” (Fig. 1B). Of the luminescence switch “OFF” and “ON” responses, the emission switch “ON” chemosensors are preferable for imaging analysis because the enhancement of the luminescence intensity can be easily observed by microscopy. The emission wavelengths of the chemosensors can also be shifted after the response processes (Fig. 1C) [25–27], allowing ratiometric luminescence detection and imaging of targeted analytes with the potential for precise and quantitative analyses. The changes of emission signals generally correspond to the concentrations of the targeted analyte and thus can be recorded for the analyte’s determination of abundance by luminescence spectroscopes and/or microscopes. Because of the unique advantages of luminescence bioassays and imaging, enormous efforts have been devoted to the development of luminescent chemosensors for the detection of a variety of analytes in complicated biological and environmental systems in the past few decades.

As shown in Fig. 1, luminescence chemosensors generally consist of three parts, including the luminophore, response unit and spacer, which links the luminophore and response unit. Of all luminophores that are being used for the development of chemosensors, fluorescent organic dyes are most widely investigated because of their high quantum yields (ϕ) and easy modification of chemical structures [28]. Lanthanide chelates are another family of luminophores that have been successfully employed in the development of chemosensors [29, 30]. Compared with fluorescent organic dyes, lanthanide chelate luminescence has high photostability, large Stokes shift and unique line-like emissions [29]. The prolonged lifetime of lanthanide chelates (microseconds to milliseconds) enables a background-free bioassay and imaging of targeted analytes through time-gated luminescence (TGL) measurement [28, 31]. Transition metal complexes, particularly the luminescent ruthenium(II) (Ru(II)) [32], iridium(III) (Ir(III)) [33], platinum(II) (Pt(II)), gold(I) (Au(I)) [34], rhenium(I) (Re(I)) [35] and osmium(II) (Os(II)) complexes with d^6^, d^8^ and d^10^ electron structures, have also been studied when developing chemosensors for biomolecule and ion detection and imaging [36–40]. Different from the fluorescent organic dyes that emit from excited singlet state, phosphorescence of transition metal complexes is derived from excited triplet states [41]. The excited states of these transition metal complexes are more complicated than those of fluorescent dyes and mainly include metal-to-ligand charge transfer (MLCT), intraligand charge transfer (ILCT), ligand-to-ligand charge transfer (LLCT), metal–metal-to-ligand charge transfer (MMLCT), ligand-to-metal charge transfer (LMCT), metal-to-ligand-ligand charge transfer (MLLCT) and ligand-to-metal–metal charge transfer (LMMCT) [41–43]. The excited state-mediated emission properties of transition metal complexes are varied upon the changes of the metal center, local environment and particularly chemical structure of ligands, enabling transition metal complexes to be designed as the chemosensors through modulating these parameters [43].

**Fig. 2:**
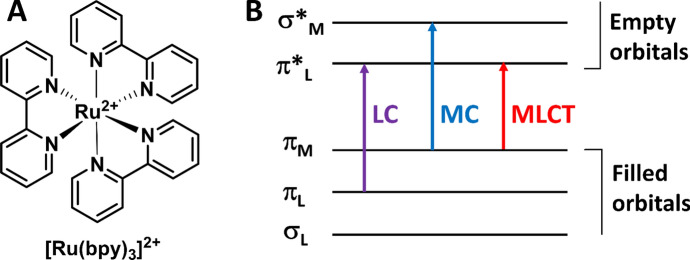
Molecular structure of [Ru(bpy)_3_]^2+^ prototype complex (**A**). Ru(II) polypyridine complexes’ molecular orbital diagram and the corresponding LC, MC and MLCT electronic transitions (**B**)

Of all transition metal complex-based luminophores, Ru(II) polypyridine complex, particularly the prototype of the Ru complex ([Ru(bpy)_3_]^2+^ (bpy: 2,2′-bipyridine)) (Fig. 2A), has been one of the most popular molecules and widely investigated in the past few decades [44]. Ru(II) polypyridine complexes have octahedral symmetry with three kinds of electronic transitions, including metal centered (MC), ligand centered (LC) and MLCT [41]. As shown in Fig. 2B, in this octahedral symmetry of Ru(II) complex, MC excited states are obtained for an electron transition from π_M_ to σ*_M_ orbitals, LC excited states are formed through an electron transition from π_L_ to π*_L_, and MLCT excited states are produced by promotion of an electron from π_M_ metal orbital to π*_L_ ligand orbitals [45]. The lowest excited state MC can decay to the ground state through a fast radiationless process. In contrast, the lowest excited states LC and MLCT undergo radiative deactivation to the ground state, thus exhibiting intense luminescence at room temperature in a rigid matrix and fluid solution, respectively [41]. Consequently, the lowest excited state is ^3^MLCT for most luminescent Ru(II) polypyridine complexes in solution. Upon the excitation at about 450 nm (spin-allowed ^1^MLCT), the lowest spin-forbidden ^3^MLCT excited state is obtained after a fast intersystem crossing process and then emits orange to near infrared emission [46]. The ^3^MLCT-based emission of Ru(II) polypyridine complexes displays unique photochemical and photophysical properties, including large Stokes shift (about 150 nm), prolonged luminescence lifetime (hundreds of nanoseconds to microseconds level), high photostability and brightness by visible light excitation [41, 47].

The emission of Ru(II) complexes, including luminescence intensity and lifetime, can be fine-modulated by modifying the chemical structure of ligands, allowing the Ru(II) complexes to be used for the development of chemosensors for biomolecule and ion detection. As described in early review articles [42, 43, 48, 49], the response mechanisms of luminescent Ru(II) complex chemosensors for target analytes mainly include (1) photo-induced electron transfer (PeT), in which the Ru(II) polypyridine complexes are ideal electron donors and acceptors, (2) Förster resonance energy transfer (FRET), in which the Ru(II) complexes can serve as the energy donor and acceptor [50–53] for the energy transfer (ET), (3) distortion of ligand and the octahedral symmetry after binding/reaction with the target analyte and (4) changes of the local environment upon the analyte’s binding. In addition to the luminescence response of Ru(II) complex-based chemosensors to the analyte [40, 54, 55], other prerequisites for this family of chemosensors to be applied in bioassay and imaging include (1) capability of analyte determination in aqueous solution, (2) high sensitivity and selectivity, which allow the chemosensors to be used for targeted analyte detection even at extremely low concentration without non-specific binding of interference species, (3) low cytotoxicity, enabling targeted analyte determination and imaging with minimum perturbation to the native micro-environment and (4) high cell membrane permeability to ensure the chemosensors are easily internalized into biological tissues.

**Fig. 3:**
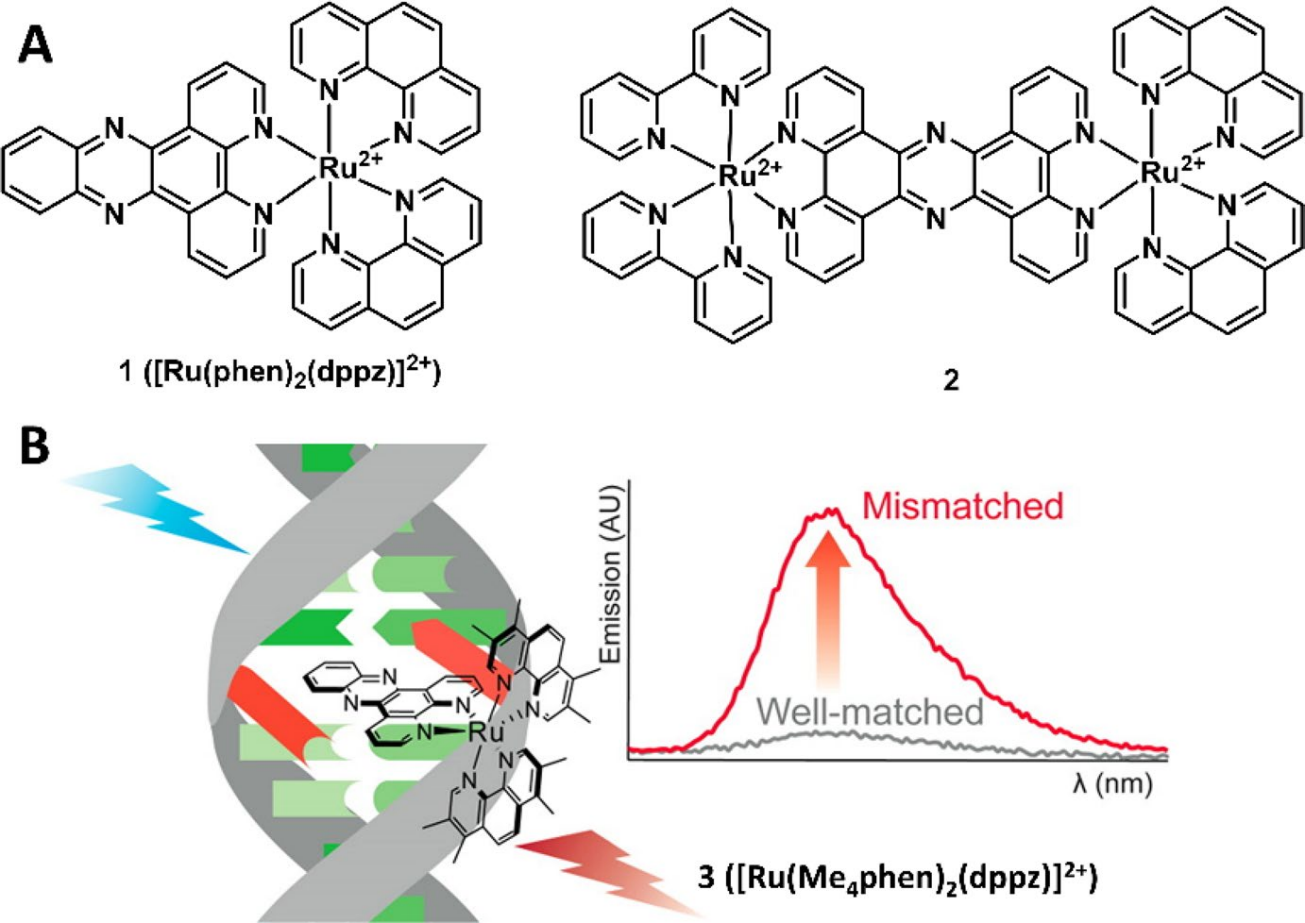
Molecular structures of examples of Ru(II) complexes **1** ([Ru(phen)_2_(dppz)]^2+^) and **2** for DNA determination and imaging (**A**) and complex **3** ([Ru(Me_4_phen)_2_(dppz)]^2+^) for detection of DNA mismatches. The luminescence spectra represent the emission of complex **3** with well-matched and mismatched DNA. Adapted with permission from Ref. [67]. Copyright 2016 American Chemical Society

The last few years have witnessed a huge leap forward in the development of Ru(II) complexes as the chemosensors for the detection of various analytes. Based on the above-described response mechanisms, hundreds of Ru(II) complex chemosensors for colorimetric and luminescent determination of anions, metal ions, small biomolecules and biomacromolecule have been available [42, 43]. For example, the triplet nature of the emission state with long lifetime of Ru(II) complexes allows them to be used for oxygen sensing by monitoring the changes of their luminescence intensity and lifetime [56–58]. Some of the Ru(II) complexes have also been revealed to be nucleic acid sensitive [59, 60], thus serving as a “light switch” for DNA detection [61]. The Ru(II) complexes with dipyridophenazine (dppz) ligands (e.g., complex **1** ([Ru(phen)_2_(dppz)]^2+^) are particularly interesting (Fig. 3A) [61]. These complexes are non-luminescent (undetectably small quantum yield) in water, while the emission intensity is significantly increased when bound to DNA [62, 63], which allows the Ru(II) complexes (e.g., complex **2**) to be used for imaging of DNA structure and related mitosis progression (Fig. 3A). Subsequent research has also revealed that Ru(II) complexes (e.g., complex **3**) can be used for the determination of DNA mismatches and RNA (Fig. 3B) [64–67]. Since the investigation of cellular uptake and imaging of the Ru(II) complex by Barton’s group [68, 69], the application of Ru(II) complex chemosensors for sensing and imaging of intracellular biomolecules and ions has increasingly attracted interest in recent years.

In this chapter, we wish to summarize recent examples of Ru(II) complex chemosensors for the detection of small biomolecules and ions in aqueous solution, with a particular focus on binding/reaction-based chemosensors for the investigation of intracellular analytes’ evolution through luminescence imaging. Specifically, the chemosensors for the determination of reactive oxygen/nitrogen/carbonyl species (ROS/RNS/RCS), biothiols, amino acids, pH, metal ions and anions are summarized, followed by the discussion of these chemosensors for luminescence bioimaging. The advances, challenges and future research directions in the development of Ru(II) complex-based chemosensors will also be discussed.

## Ru(II) Complex Chemosensors for Anions

In various chemical and biological processes, anions play important roles in the body, such as blood pressure stabilization, blood purification, sugar level reduction, respiration and fatigue recovery. For anion sensing, the development of Ru(II) complex-based chemosensors has attracted enormous attention in the past few decades [70–72]. By virtue of their abundant photo-physical and chemical properties, hundreds of Ru(II) complexes have been designed and synthesized for the detection of various anions, such as fluoride (F^−^) [73–76], acetate (CH_3_COO^−^) [77], cyanide (CN^−^)[78], phosphate (H_2_PO_4_^−^) [79–81], chloride (Cl^−^) and bromide (Br^−^). Similar to the design of other anion receptors [82], most Ru(II) complex-based chemosensors are designed using the following three response mechanisms, including (1) the binding of the Ru(II) complex’s recognition unit with anions via hydrogen bonding and deprotonation [83], electrostatic and Lewis acid–base interactions [84], (2) specific reactions of the Ru(II) complex’s recognition unit with anions [85] and (3) displacement of metal ions from heterobimetallic Ru(II) complex [86]. In the following section, the Ru(II) complex-based chemosensors for anions will be discussed according to their different response mechanisms.

### Response Based on Hydrogen Bonding, Electrostatic and Lewis Acid-Base Interactions

Although most Ru(II) complex-based anion chemosensors are developed through the mechanism of hydrogen bonding and electrostatic and Lewis acid-base interactions, the colorimetric and luminescent response of these Ru(II) complexes to anions can only be obtained in organic solvents, including acetonitrile (CH_3_CN) and dimethyl sulfoxide (DMSO) [87, 88]. This is mainly because the hydrogen bonding, electrostatic and Lewis acid-base interactions are significantly inhibited by the water molecules and other anions in the buffer solution [89]. This sub-section will discuss some examples of Ru(II) complex chemosensors for determination of anions in aqueous solution [90–92].

**Fig. 4:**
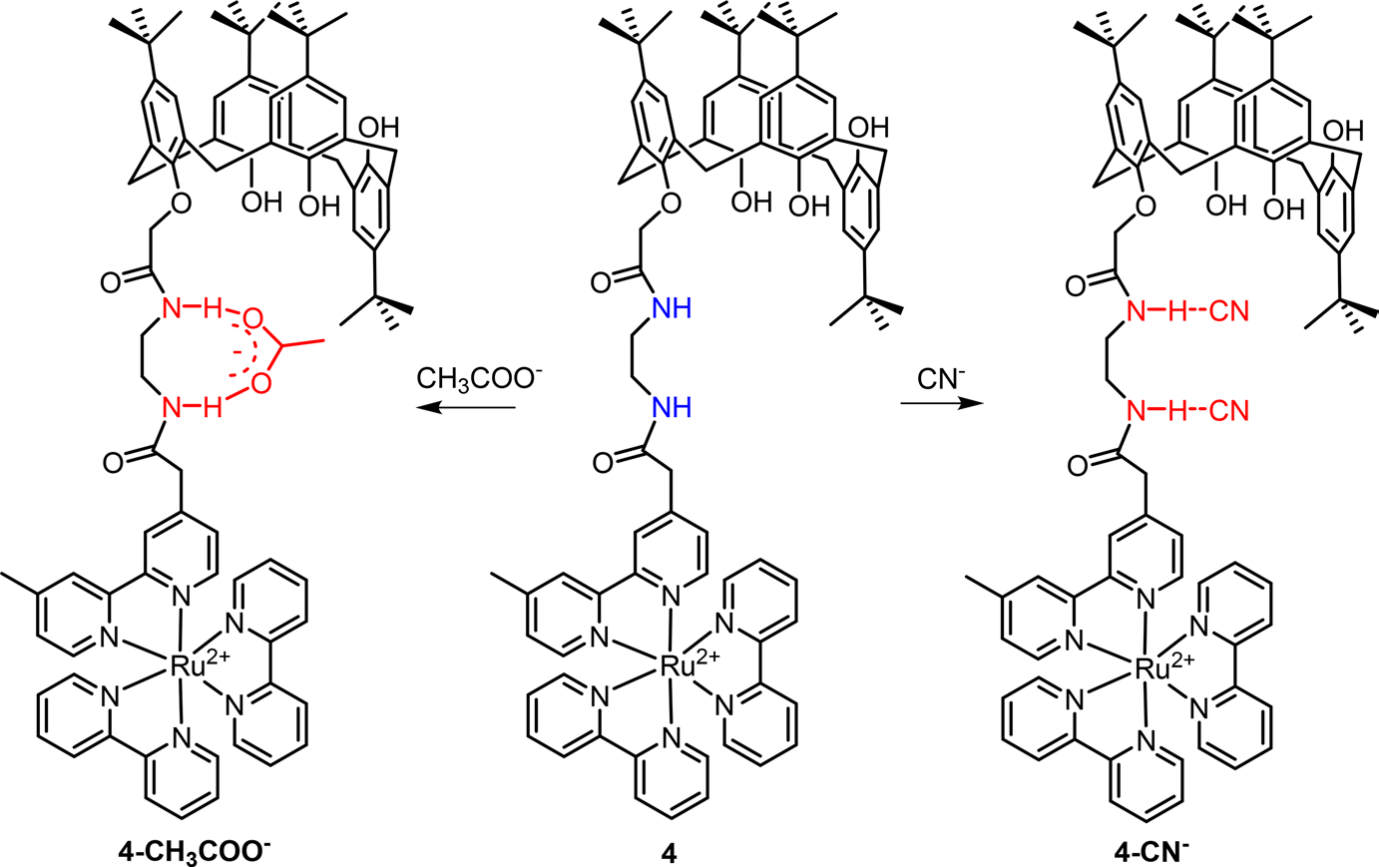
Molecular structure of Ru(II) complex **4** and its response mechanism to CN^−^ and CH_3_COO^−^

Through modification of the [Ru(bpy)_3_]^2+^ with amide containing a calixarene moiety (binding site), Maity et al. reported a Ru(II) complex (**4**) for CN^−^ and CH_3_COO^−^ determination (Fig. 4) [93]. Titration of complex **4** with CN^−^ and CH_3_COO^−^ in H_2_O–CH_3_CN (95:5) resulted in a remarkable luminescence quenching and enhancement, respectively. The different response mechanisms of complex **4** to CN^−^ and CH_3_COO^−^ were investigated by ^1^H NMR spectra. The CN^−^ can bind to each amide N–H and leads to the deprotonation to form HCN. The electron density on the bpy ligand increased, and thus the intramolecular quenching was enhanced. In contrast, the weak interaction of bidentate CH_3_COO^−^ with two N–H protons led to the formation of electron delocalization, in which the CH_3_COO^−^ pulls the electron density to itself and thus decreases the intramolecular quenching. The luminescence response of complex **4** to CN^−^ showed a LoD of 70 ppb.

**Fig. 5:**
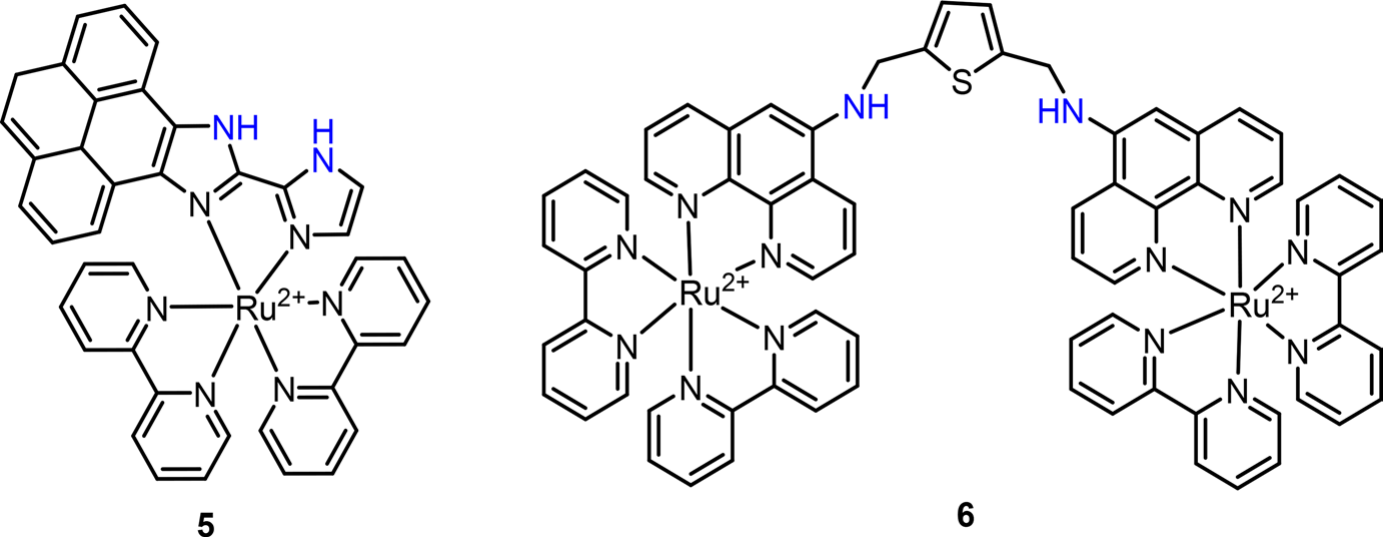
Molecular structures of Ru(II) complexes **5** and **6** as the chemosensors for CN^−^ and SCN^−^, respectively

Mardanya and co-workers described a Ru(II) complex (**5**) with pyrene-biimidazole ligand as a chemosensor for highly selective CN^−^ detection in both CH_3_CN and aqueous media (Fig. 5) [94]. The imidazole N–H protons of the coordinated ligand were found to be highly acidic with *pK*a_1_ = 5.09 and *pK*a_2_ = 8.95. Deprotonation of these two N–Hs was found through hydrogen bonding interaction with CN^−^, leading to the increase of electron density of the metal center. As a result, red shift of absorption and quenching of emission were obtained for complex **5** after CN^−^ binding. Detection limit of complex **5** to CN^−^ was determined to be 5.24 × 10^–9^ and 4.67 × 10^–9^ M for colorimetric and luminescent analyses, respectively. A similar hydrogen bonding-based interaction has been employed for the development of Ru(II) complex **6** for selective detection of thiocyanate (SCN^−^) (Fig. 5) [95]. In complex **6**, the SCN^−^ interacts with N–H through a 1:1 binding mode, which hinders the photo-induced electron transfer (PeT) between the long pair electron of the N atom and the Ru(II) complex. The increase of luminescence intensity was thus recorded for SCN^−^ detection in CH_3_CN-HEPES buffer solution (1:1, v/v, pH = 7.2).

**Fig. 6:**
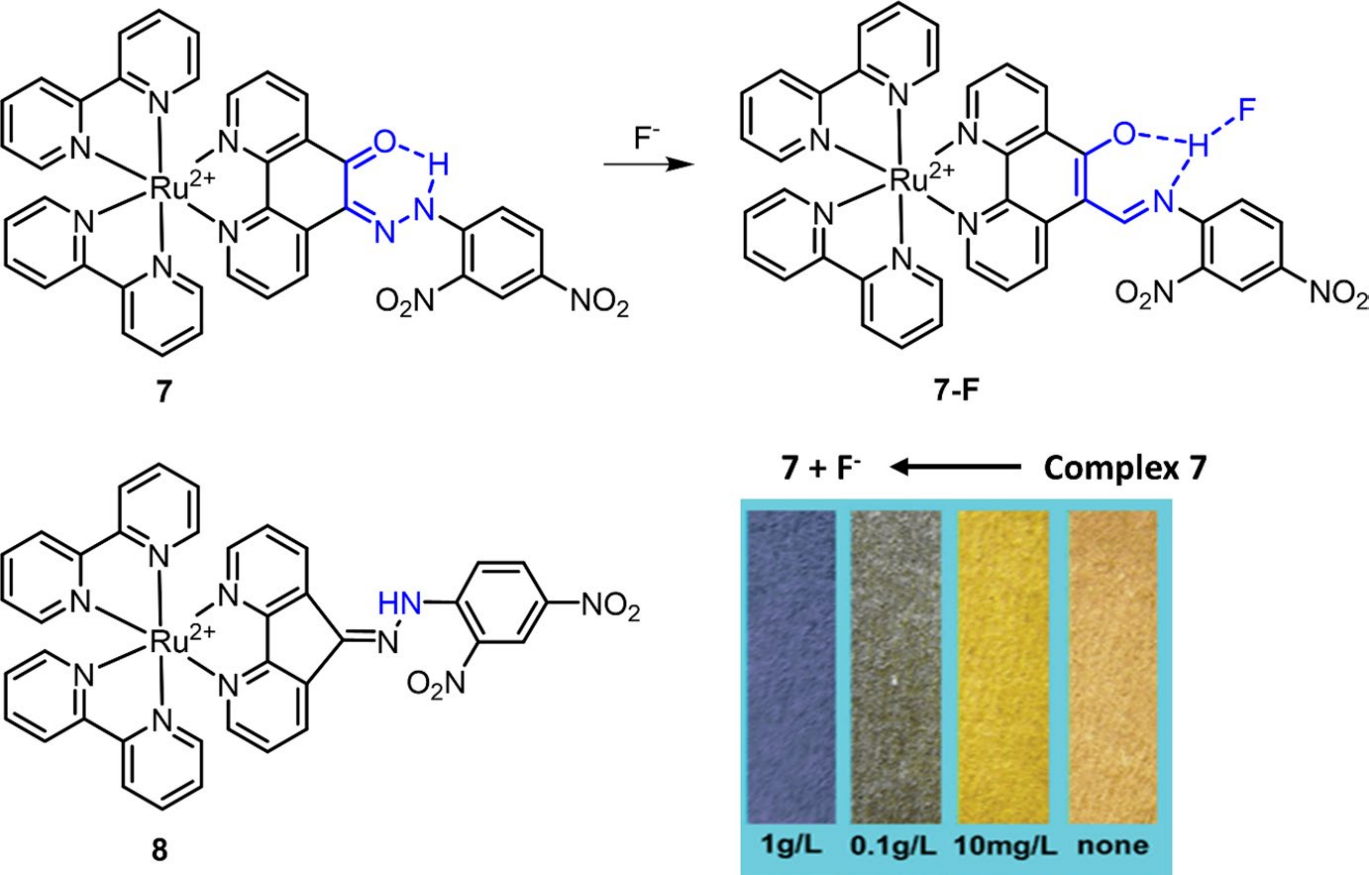
Molecular structure of Ru(II) complexes **7** and **8** as chemosensors for F^−^. The test paper prepared by complex **7** was then used for F^−^ detection in aqueous solution. Adapted with permission from Ref. [96]. Copyright 2006 Royal Society of Chemistry

In an early study, Lin et al. reported a Ru(II) complex (**7**) for highly selective detection of F^−^ in water by naked eye and luminescence (Fig. 6) [96]. Complex **7** was developed by incorporating a Schiff-base ligand with two bpy ligands. In the presence of F^−^, the conversion of quinonehydrazone moiety to azophenol could occur, resulting in a remarkable red shift of absorption spectra from 475 to 580 nm and a solution color change from orange to blue-violet. The binding of F^−^ also led to the increase of luminescence intensity at 630 nm. Although the spectrometric responses were measured in CH_3_CN, the test paper prepared by staining of complex **7** also showed color changes in aqueous solution. In a later study, the same group modified the 1,10-phenanthroline-5,6-dione ligand to produce complex **8** for F^−^ detection (Fig. 6) [97]. In the presence of F^−^, a similar red shift of absorption spectra (from 467 to 580 nm), color change (from yellow to magenta) and luminescence enhancement were obtained, which were attributed to the F^−^-mediated hydrogen bonding and deprotonation of N–H. The test papers were also prepared for F^−^ detection in aqueous solution with ten times higher sensitivity (LoD = 1 ppm) than complex **7**.

### Response Based on Specific Reactions

**Fig. 7:**
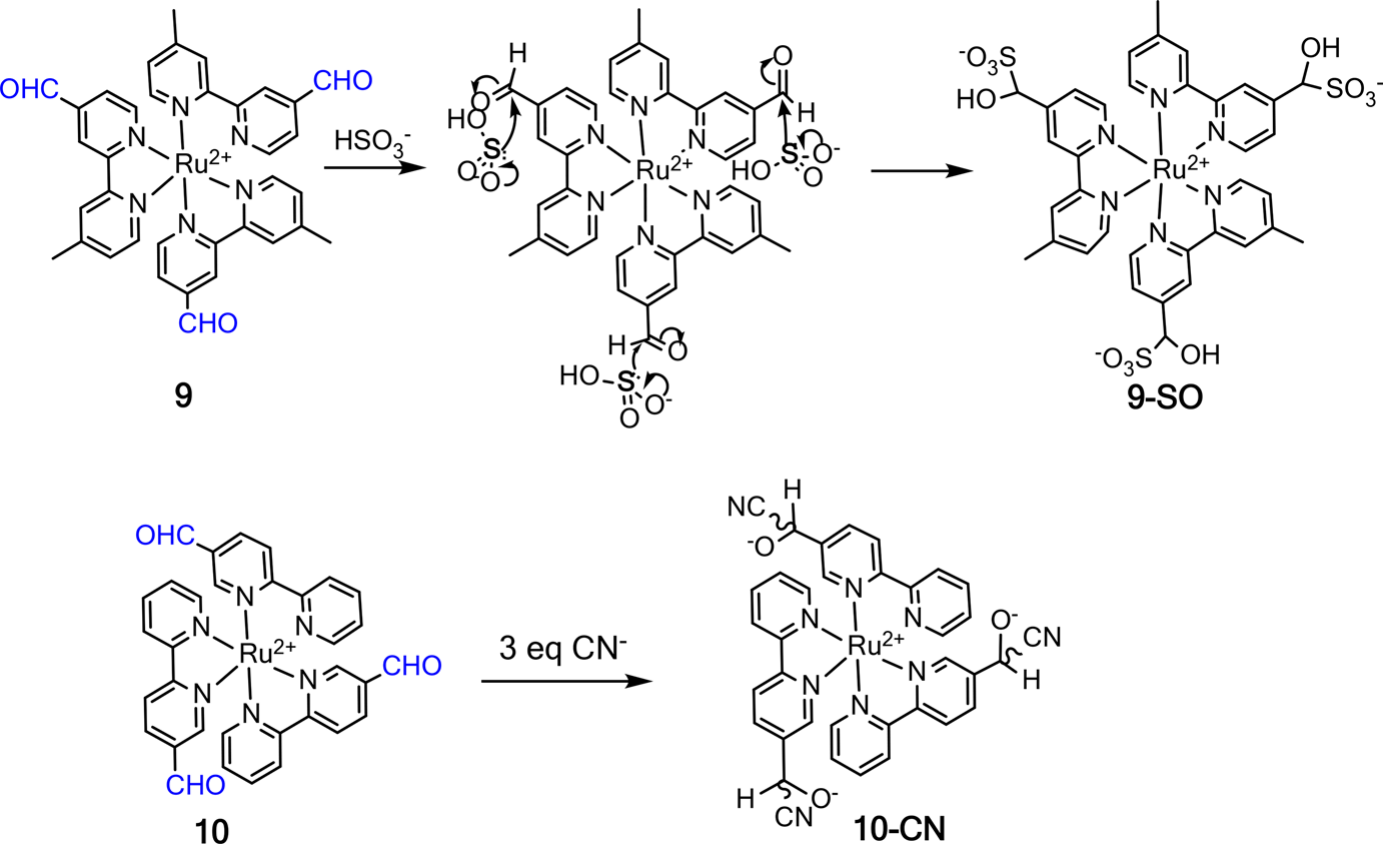
Molecular structures of Ru(II) complexes **9** and **10** and their reactions with HSO_3_^−^ and CN^−^, respectively

Compared with the above-discussed response mechanism of hydrogen bonding and Lewis acid-base interactions, the chemosensors developed by the mechanism of chemical reaction have high sensitivity and selectivity [78, 98, 99]. The interference from water is also minimized because specific chemical reactions are involved in the sensing mechanism of these chemosenosors. Aldehyde is a strong electron-withdrawing group that can quench the MLCT emission of Ru(II) complex. In a previous study, a Ru(II) complex (**9**, [Ru(CHO-bpy)_3_]^2+^) with three aldehyde functionalized bpy ligands was designed and synthesized by Zhang et al. as the chemosensor for biothiol (cysteine-Cys and homocysteine-Hcy) detection in DMSO-HEPES buffer [100]. In a later study, Zhang et al. found that the nucleophilic addition of aldehyde can also be triggered by hydrogen sulfite (HSO_3_^−^) in acidic buffer [24], thus allowing complex **9** to be used as a chemosensor for HSO_3_^−^ detection in phosphate-buffered saline (PBS) buffer (50 mM, pH = 5) (Fig. 7) [101]. The reaction of HSO_3_^−^ and complex **9** led to the formation of **9-SO**, accompanied by an increase of luminescence at 620 nm. The result of HSO_3_^−^ detection in wine and sugar samples showed that complex **9** has good precision and accuracy for HSO_3_^−^ in food samples. Using 5-formyl-2,2′-bipyridine as the ligand, Zhu et al. prepared a Ru(II) complex (**10**) as the luminescence chemosensor for CN^**−**^ detection (Fig. 7) [102]. Similar to [Ru(CHO-bpy)_3_]^2+^, complex **10** in CH_3_CN-H_2_O (6:4) showed weak emission at 700 nm. The nucleophilic attack by CN^−^ led to the formation of **10-CN**, accompanied by a blue shift and increase of emission at 618 nm. Complex **10** with a detection limit of 0.75 μM was then used for the preparation of a test paper for naked eye CN^−^ detection.

**Fig. 8:**
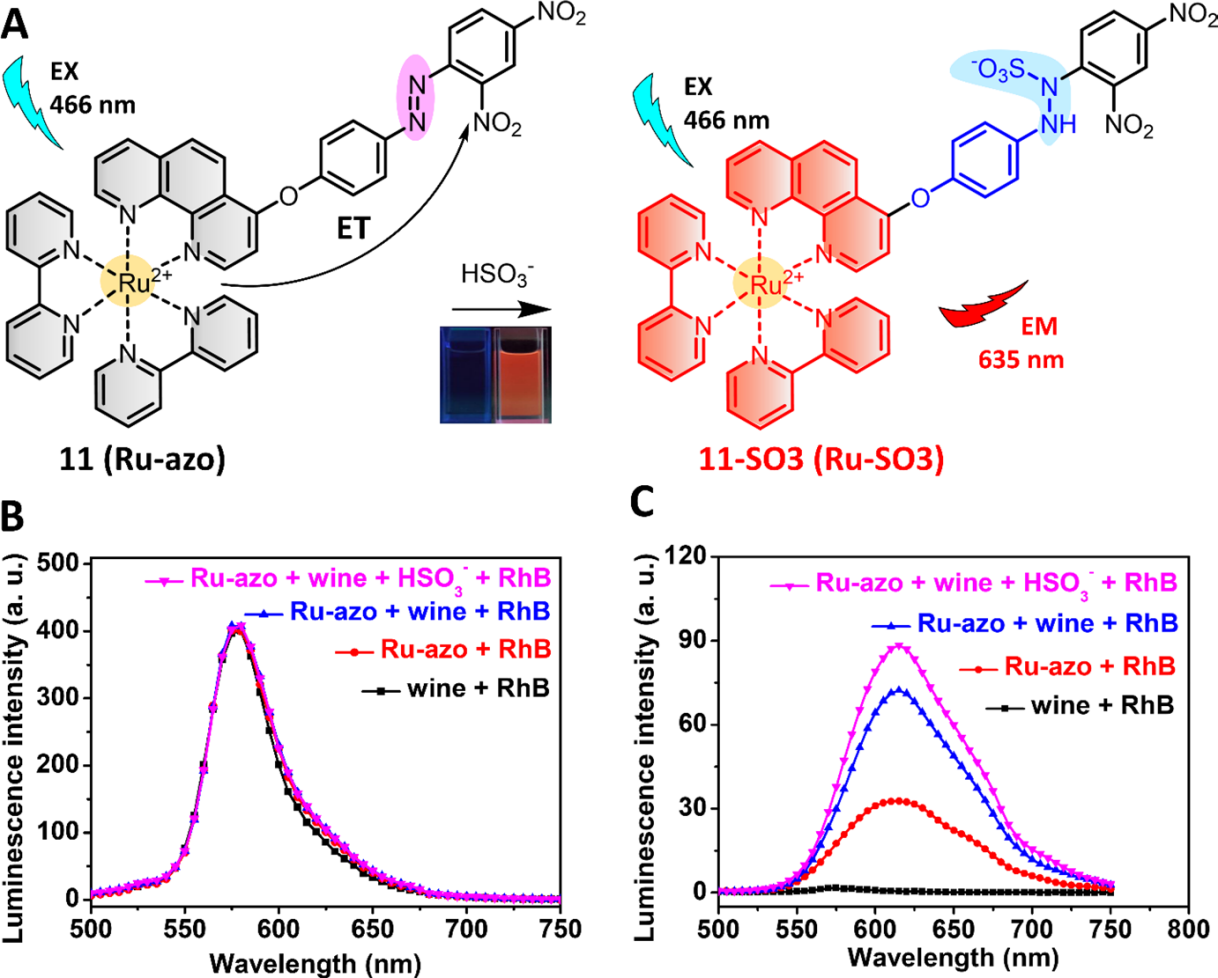
Molecular structure of Ru(II) complex **11** and its reaction with HSO_3_^−^ (**A**). Detection of HSO_3_^−^ in rhodamine (RhB)-contaminated wine samples by steady-state luminescence (**B**) and TGL (**C**) analyses. Adapted with permission from Ref. [85]. Copyright 2020 Royal Society of Chemistry

In addition to aldehyde, nucleophilic addition between the azo (N = N) group and HSO_3_^−^ has recently been exploited for the development of chemosensors for HSO_3_^−^ detection (Fig. 8A) [24, 85]. Owing to the PeT from the Ru(II) center to the attached azo-2,4-dinitrobenzene (DNB), Ru(II) complex **11** (Ru-azo) exhibited weak emission in 25 mM PBS buffer of pH 7.4. The HSO_3_^−^ triggered reaction with the azo group led to the formation of **11**-SO3 (Ru-SO3), accompanied by an enhancement in luminescence at 635 nm. More interestingly, complex **11** has a long emission lifetime of 258 ns, which enabled its use for background-free luminescence detection of HSO_3_^−^ through a TGL mode. In a wine sample containing rhodamine (spiked as the artificial background signal), steady-state luminescence analysis of HSO_3_^−^ failed (Fig. 8B). TGL analysis eliminated the background signals and allowed for HSO_3_^−^ detection with high accuracy and precision (Fig. 8C).

### Response Based on Displacement of Metal Ions

**Fig. 9:**
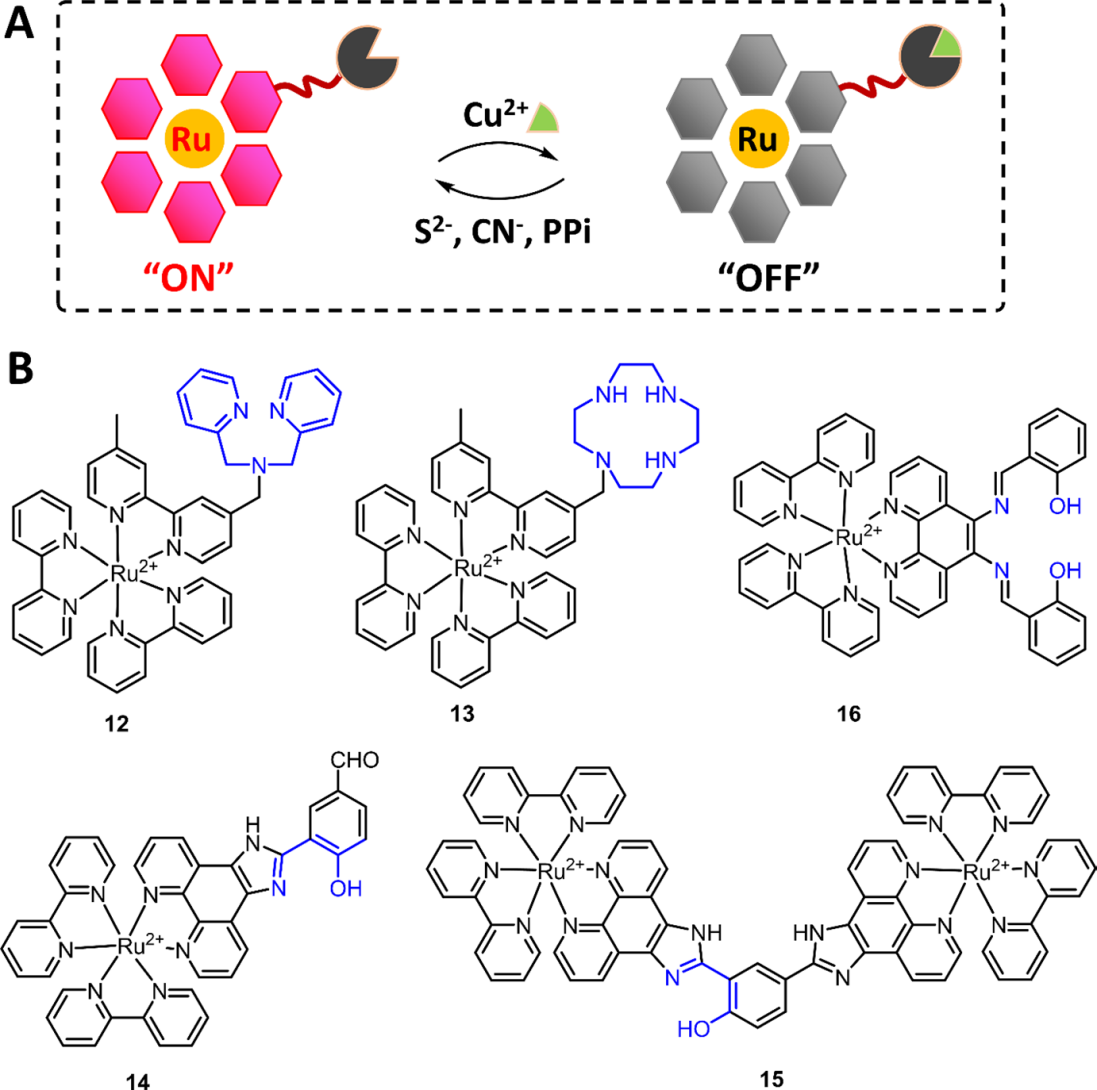
The principle of displacement-based Ru(II) complex chemosensors for anion detection (**A**) and the molecular structures of Ru(II) complexes **12**–**16**

By modifying the ligands with additional coordination sites, the produced Ru(II) complexes are capable of binding to other metal ions, such as Cu^2+^, Co^2+^, Zn^2+^ and Hg^2+^, to form heterobimetallic complexes. The heterobimetallic Ru(II) complexes thus can be used as chemosensors for anion sensing through a displacement approach. Different from the hydrogen bonding-based anion sensing approach, the displacement-based response mechanism also allowed the chemosensors to be used for anion detection in water because the water molecules are not involved in the displacement processes. Among various heterobimetallic Ru(II) complexes, the one with Cu^2+^ (Ru(II)–Cu(II)) has been widely studied because Ru(II) complex luminescence can be quenched by Cu^2+^ binding through electron and energy transfers. Then, Cu^2+^ can be displaced in the presence of several anions, including sulfide (S^2−^), CN^−^ and pyrophosphate (PPi) (Fig. 9A).

By coupling di(2-picolyl)amine (DPA) to one bpy ligand, Zhang et al. synthesized a Ru(II) complex (**12**) and then demonstrated the corresponding heterobimetallic Ru(II)–Cu(II) complex as the chemosensor for S^2−^ detection (Fig. 9B) [86]. Complex **12** showed high luminescence (ϕ = 3.07%) in HEPES buffer (pH 7.2), while the luminescence was completely quenched upon binding to Cu^2+^ through a 1:1 coordination stoichiometry. In the presence of S^2−^, Cu^2+^ was then displaced to form the original complex **12**, which was accompanied by the recovery of luminescence. This heterobimetallic Ru(II)–Cu(II) complex showed high sensitivity to S^2−^ (LoD = 20.7 nM), allowing its use for S^2−^ detection in three wastewater samples. In a similar work, Li et al. modified the bpy ligand with a Cu^2+^ receptor, 1,4,7,10-tetraazacyclododecane (cyclen), and then demonstrated the Ru(II) complex (**13**) for sequential Cu^2+^ and S^2−^ detection (Fig. 9B) [103]. Compared with complex **12**, complex **13** has better selectivity to Cu^2+^ binding, while the formed **13-Cu** is not as sensitive as **12-Cu** for S^2−^ sensing.

The displacement approach has also been employed for the development of Ru(II) complex chemosensors for CN^−^. In 2017, two Ru(II) complexes (**14** and **15**) were synthesized by Zheng and co-workers, and the corresponding heterobimetallic Ru(II)–Cu(II) complexes were used for CN^−^ detection in 20 mM HEPES buffer (pH 7.2) (Fig. 9B) [104]. A recovery of luminescence was observed after the displacement of Cu^2+^ from **14-Cu** to **15-Cu** to form [Cu(CN^−^)X]^n−^ and complexes **14** and **15**. The LoD for CN^−^ was then determined to be 0.36 and 0.87 μM using **14-Cu** and **15-Cu** as the chemosensors, respectively. Similarly, Zhang et al. reported a Ru(II) complex (**16**) for PPi detection in 2018 (Fig. 9B) [105], in which one Cu^2+^ from the **16-Cu** was displaced by the addition of two PPi. The chemosensor **16-Cu** showed high sensitivity (LoD = 0.58 nM) for PPi detection in 10 mM HEPES buffer (pH 7.4).

## Ru(II) Complex Chemosensors for pH

Similar to most pH sensors, Ru(II) complex chemosensors for pH have mainly been developed through the mechanism of protonation and deprotonation of several functional groups, such as imidazole [106, 107], hydroxyl (–OH) [108–110], carboxyl (–COOH) [111], pyridine [112, 113] and others [114]. As a result of the protonation-deprotonation process, the molecular structures and the electron density distribution of Ru(II) complex are changed, leading to the variations of absorption and emission intensity/wavelength. In this section, the progress in the development of Ru(II) complex chemosensors for pH will be briefly discussed.

**Fig. 10:**
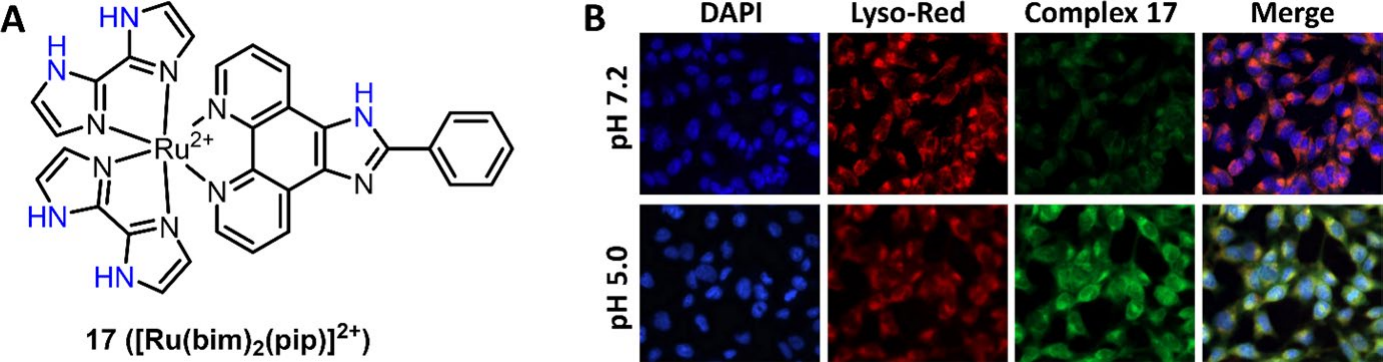
Molecular structure of pH-sensitive Ru(II) complex **17** (**A**). Luminescence co-localization imaging of U251 cells stained with complex **17** and DYPI and LysoTracker Red (**B**). Adapted with permission from Ref. [116]. Copyright 2017 Elsevier

As described above, deprotonation of N–H of Ru(II) complex **5**’s 2,2′-biimidazole ligand occurs under basic conditions or binding with CN^−^ [94]. In 2020, Tormo et al. also reported the use of imidazole-based ligand for the development of Ru(II) complex chemosensors for pH [115]. In later research, deprotonation of imidazole N–H under neutral and basic conditions was exploited by Yu et al. for the development of Ru(II) complex **17** ([Ru(bim)^2^(pip)]^2+^) for pH sensing and imaging (Fig. 10A) [116]. The increase of pH led to the deprotonation of all imidazole N–H from both bim and pip ligands, resulting in red shift of absorption spectra and decrease of MLCT emission. Complex **17** has a pKa of 4.49 and low cytotoxicity, enabling lysosome imaging in U251 cells. Luminescence images of U251 cells with complex **17** and LysoTracker Red showed good co-localization (Fig. 10B), and then the application of complex **17** in monitoring of intracellular pH changes was demonstrated by treating the U251 cells with lysosomal acidification inhibitor (bafilomycin A1).

**Fig. 11:**
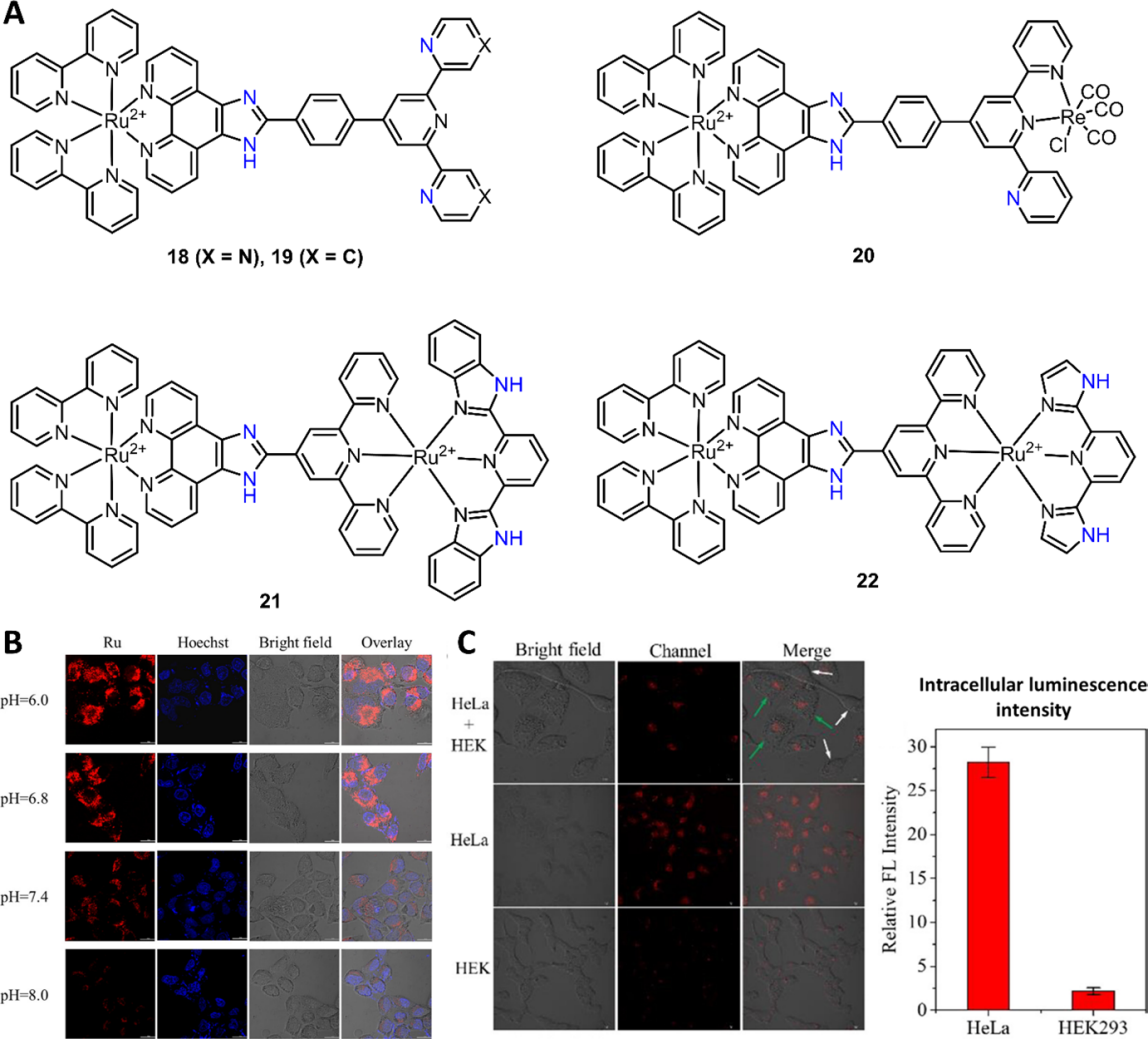
Molecular structures of pH-sensitive Ru(II) complexes **18**–**22** (**A**) and the application of complex **22** for HeLa cancer cell pH imaging (**B**) and discrimination from healthy HEK293 cells (**C**). Adapted with permission from Ref. [118]. Copyright 2020 American Chemical Society

In 2015, Zheng and coworkers synthesized two Ru(II) complexes (**18** and **19**) and investigated their absorption and luminescence responses to pH (Fig. 11A) [112]. Protonation-deprotonation process occurs on the imidazole N–H and both imidazole N–H and pyridine for complexes **18** and **19**, respectively. The two-step protonation-deprotonation processes resulted in complex **18** with pKa_1_ = 0.98 ± 0.04 and pKa_2_ = 8.34 ± 0.03, while the three-step protonation-deprotonation processes of complex **19** exhibited pKa_1_ = 1.86 ± 0.02, pKa_2_ = 3.43 ± 0.04 and pKa_3_ = 9.07 ± 0.08. Coordination of the terpyridine (tpy) ligand of complex **19** with Re(I), a heterobimetallic Ru(II)–Re(I) complex **20**, was developed by Zheng et al. in 2014 (Fig. 11A) [113]. The coordination of the tpy ligand blocked the protonation-deprotonation of one pyridine; thus, a two-step protonation-deprotonation process was obtained. The pKa_1_ and pKa2 value of complex **20** was 1.38 ± 0.03 and 6.84 ± 0.04, respectively. Interestingly, the coordination with Re(I) significantly improved the biocompatibility, allowing complex **20** to be used for luminescence imaging in HeLa cells.

Dinuclear Ru(II) complex **21** was then synthesized by Meng and co-workers in 2017 (Fig. 11A) [117]. Ru(II) complex **21** showed NIR emission at 760 nm with a large Stokes shift of 254 nm and lifetime (τ) 108.3 ± 0.4 ns. Different from complex **20**, a three-step protonation-deprotonation process was observed on imidazole N–H at the second ligand. pKa values of complex **21** changed to pKa_1_ = 1.36 ± 0.02, pKa_2_ = 5.76 ± 0.05 and pKa_3_ = 9.01 ± 0.14. Through modification of the second ligand, the same group recently reported a Ru(II) complex (**22**) as a chemosensor for pH imaging and cancer cell discrimination (Fig. 11A) [118]. Compared with complex **20**, similar photophysical properties, including intense NIR emission (~ 700 nm) and large Stokes shift (~ 200 nm) were obtained for complex **22**. More importantly, the pKa_2_ value of complex **22** was determined to be 7.87, which is closer to the physiological value (i.e., 7.0–7.4). This allowed complex **22** to be used for luminescence imaging of intracellular pH in lysosomes (Fig. 11B). Moreover, imaging of HeLa cells showed about 13-fold higher intensity than HEK293 cells (Fig. 11C), demonstrating the “distinguishing” ability of complex **22** to identify the tumor and healthy cells.

## Ru(II) Complex Chemosensors for Metal Ions

In addition to the anions, metal ions also play important roles in biological and environmental systems. Some metal ions, such as Cu^2+^, Fe^3+^ and Zn^2+^, are essential elements in the human body, while the metal ions, such as Hg^2+^, Cd^2+^ and Cr^3+^, are highly toxic, causing several problems for biological and environmental systems [119]. To detect these metal ions in biological and environmental systems, a number of Ru(II) complex chemosensors have been developed in the past few decades. In this section, the progress in the development of chemosensors for Cu^2+^, Hg^2+^ [120] and others will be discussed according to the types of ions.

### Ru(II) Complex Chemosensors for Cu^2+^

**Fig. 12:**
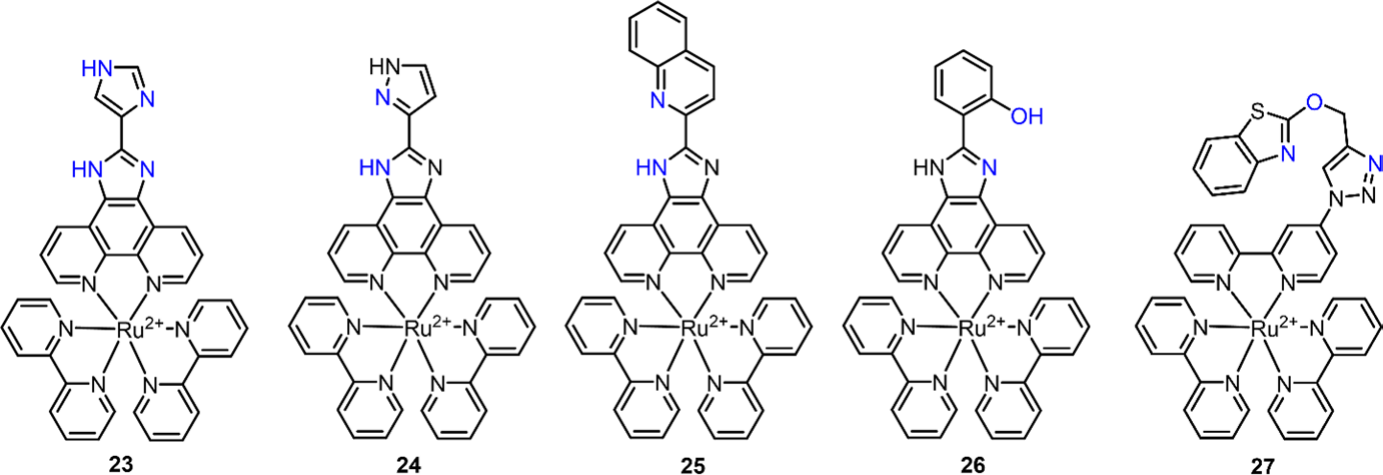
Molecular structure of Ru(II) complexes **23**–**27** as the chemosensors for Cu^2+^

As described above, the binding of Ru(II) complexes with Cu^2+^ could lead to the quenching of their luminescence through an excited-state electron transfer or energy transfer mechanism [86, 104, 105]. By virtue of this mechanism, a series of Ru(II) complexes have been developed as luminescence “ON–OFF” chemosensors for Cu^2+^ determination and imaging (Fig. 12). Complex **17** with biimidazole ligands showed good performance in pH sensing with the protonation-deprotonation mechanism [116]. In a recent study, the biimidazole-coupled phen ligand was employed as the binding site for the development of Ru(II) complex chemosensor (**23**) for Cu^2+^ detection by Li and co-workers [121]. The coordination (1:1 bonding ratio) of Cu^2+^ with complex **23**’s biimidazole led to the formation of a stable cyclic structure. The quenched emission of complex **23** showed a linearity with Cu^2+^ concentration in the range of 0.25–12 μM, and the LoD was 83.3 nM. The application of complex **23** was then demonstrated by Cu^2+^ detection in tap and lake water samples and imaging in A549 cells. For simple modification of imidazole to pyrazol, Cu^2+^ “ON–OFF” Ru(II) complex **24** was then reported by Xia and colleagues [122]. Different from complex **23**, complex **24** coordinated with Cu^2+^ through a 1:2 stoichiometry. This complex showed higher sensitivity to Cu^2+^ (LoD = 17.8 nM) compared with complex **23**. The test paper was then prepared for Cu^2+^ detection in river water samples. Interestingly, complex **25** with quinoline substitution showed a 1:1 stoichiometry when binding to Cu^2+^ in HEPES buffer (LoD = 50.67 nM) [123]. The test paper was also prepared using complex **25** as the chemosensor for Cu^2+^ detection. In another study, Zhang et al. reported a Ru(II) complex **26** for Cu^2+^ detection in aqueous solution and imaging in live pea aphids (LoD = 244 nM) [124]. Complex **26** was developed through coordination with two phen ligands and one 2-(2-hydroxyphenyl) imidazo[4,5-f][1,10]phenanthroline. The Cu^2+^ binding with 2-hydroxyphenyl imidazo through a 1:1 stoichiometry quenched complex **26**’s emission. Similar to Cu^2+^-coordinated complexes **23** and **25**, the Cu^2+^ can be displaced by the addition of EDTA (ethylene diamine tetraacetic acid), which allows the reset of the chemosensors for further detection of Cu^2+^.

In addition to imidazole, some other groups, such as DPA [86, 125–128], 1,8-naphthyridine [129], 1,3-benzothiazole [130], carboxyl [131] and others [132], have also been utilized as the response units for the development of Ru(II) complex chemosensors for Cu^2+^. For example, complex **27** reported by Ramachandran and colleagues was capable of detecting phosphate anions through C–H-anion interaction and Cu^2+^ through coordination with triazole, benzothiazole and the “O” linker, respectively [130] (Fig. 12). The application of this chemosensor (**27**) for Cu^2+^ detection (LoD = 700 nM) and imaging was also demonstrated by luminescence Cu^2+^ imaging in MCF-7 cells.

**Fig. 13:**
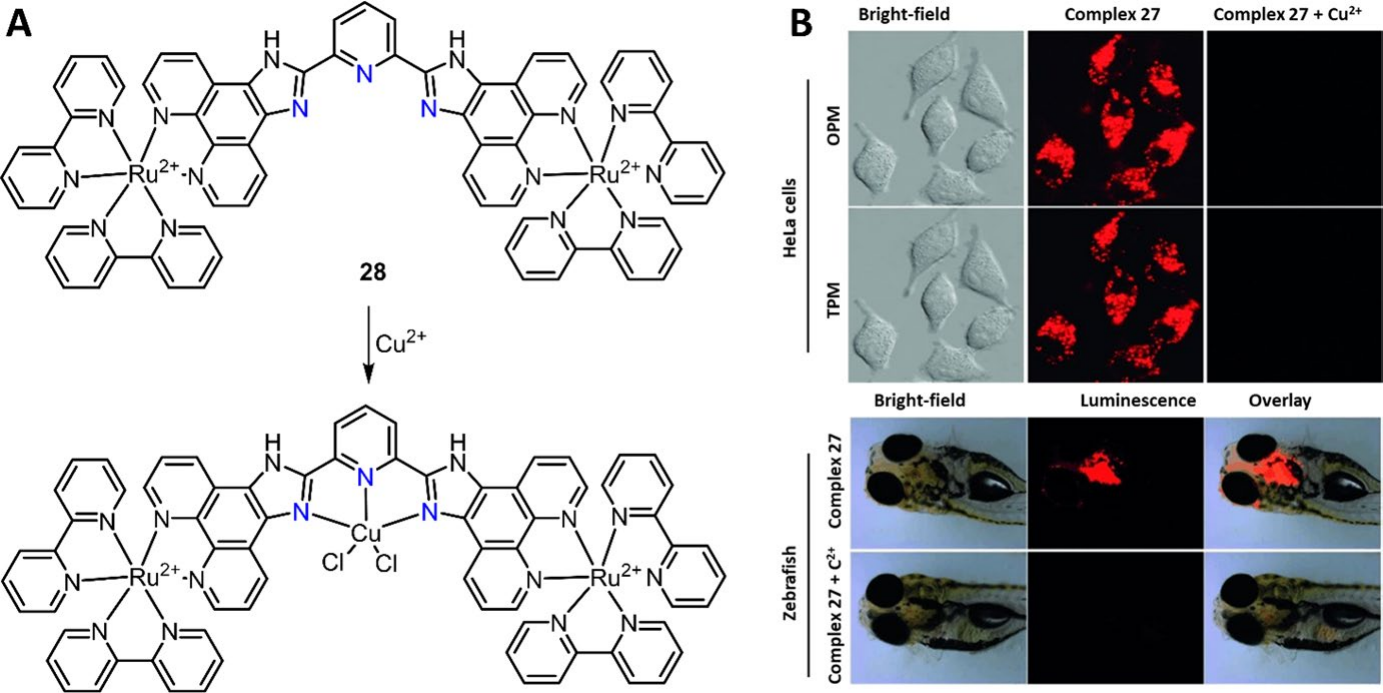
Molecular structure of Ru(II) complex **28** and its response mechanism to Cu^2+^ (**A**). One-photon microscopy (OPM) and two-photon microscopy (TPM) imaging of Cu^2+^ in cells and zebrafish (**B**). Adapted with permission from Ref. [134]. Copyright 2013 Wiley

Similar to complex **15**, the pyridine “linker” of dinuclear Ru(II) complex **28** has also been developed as the chemosensor for Cu^2+^ detection in H_2_O/CH_3_CN (1:1, v/v) [133], in HEPES buffer solution (10 mM, pH 7.4) (Fig. 13A) [134]. Complex **28** showed high luminescence (ϕ = 0.06) at 600 nm, while the Ru(II) complex’s MLCT emission was quenched after coordination of Cu^2+^ with imidazole and pyridine. This chemosensor showed high sensitivity (LoD = 33.3 nM) and selectivity, reversibility (in the presence of EDTA) and good biocompatibility and cell membrane permeability, enabling it to be used for luminescence imaging. In addition, the Ru(II) complex’s large two-photon absorption (TPA) cross-section enabled complex **28** to be used for TP imaging of Cu^2+^ in HeLa cells and zebrafish (Fig. 13B).

**Fig. 14:**
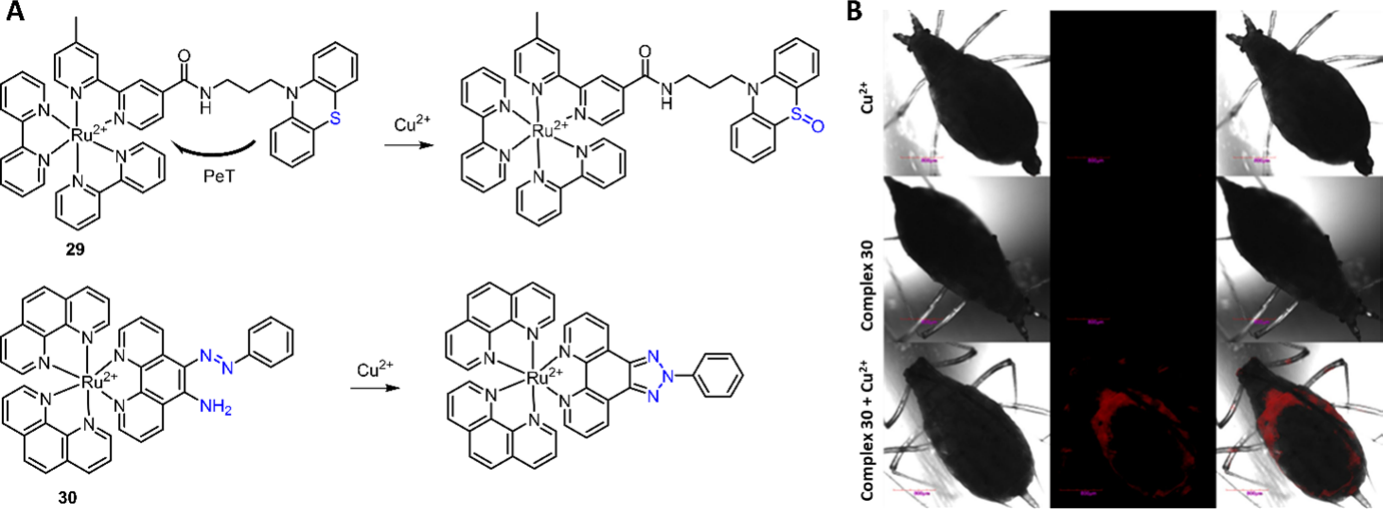
Molecular structure of Ru(II) complexes **29**, **30** and their response reaction with Cu^2+^ (**A**). The application of Ru(II) complex **30** for Cu^2+^ imaging in pea aphids. Adapted with permission from Ref. [136]. Copyright 2015 Springer Nature

Although “OFF–ON” luminescence response chemosensors feature high sensitivity and selectivity, and excellent performance in luminescent bioimaging, the development of turn “ON” response chemosensors remains a challenge due to the intrinsic luminescence quenching property of Cu^2+^. In 2009, a phenothiazine-coupled Ru(II) complex **29** was developed by Ajayakumar as the luminescence turn “ON” chemosensor for Cu^2+^ detection (Fig. 14A) [135]. Complex **29** was almost non-luminescent (ϕ = 0.0035 in CH_3_CN) because of the PeT from electron-rich phenothiazine to the Ru(II) center. In the presence of Cu^2+^, the oxidation of phenothiazine to phenothiazine-5-oxide inhibited the PeT process, and thus the emission of complex **29** was switched “ON.” In another research, Zhang et al. reported a Ru(II) complex “OFF–ON” luminescence chemosensor **30** for Cu^2+^ detection and imaging (Fig. 14A) [136]. Complex **30** with an *o*-(phenylazo)aniline structure showed weak luminescence in HEPES buffer solution (20 mM, pH 7.4). Cu^2+^-mediated oxidative cyclization led to > 80-fold enhancement in luminescence at 599 nm. The large enhancement in luminescence allowed complex **30** for highly sensitive (LoD = 4.42 nM) and selective detection of Cu^2+^ in buffer and imaging of Cu^2+^ in pea aphids (Fig. 14B).

Despite the quenching of most Ru(II) complexes’ emission by Cu^2+^ binding, the heterobimetallic Ru(II)–Cu(II) complexes provided an excellent platform for further development of “OFF–ON” response chemosensors for the detection of various analytes, such as anions [137], adenosine triphosphate (ATP) [138], amino acids [139, 140], redox biology [128] and other metal ions [141]. For example, in 2012, Wang et al. reported a Ru(II) complex for sequential detection of Cu^2+^ and Cr^3+^ in aqueous solution [141]. The coordination of a complex with Cu^2+^ produced the non-luminescent Ru(II)–Cu(II) complex, and this heterobimetallic complex showed high selectivity to Cr^3+^ in NaOAc-HOAc buffer (pH 5.6). A high sensitivity of this chemosensor to Cr^3+^ was also obtained with a LoD 66 nM.

### Ru(II) Complex Chemosensors for Hg^2+^

Because of the high binding affinity of S atom with Hg^2+^, a series of S atom-bearing Ru(II) complexes have been developed as the chemosensors for Hg^2+^ detection. Previous research has revealed the colorimetric response of N-719 to Hg^2+^, in which the absorption spectra of N-719 were blue-shifted after binding of Hg^2+^ [142, 143]. The N-719 functionalized upconversion nanoparticles (UCNPs) were then prepared, and the application of the ratiometric upconversion luminescence (UCL) nanosensor for Hg^2+^ detection and imaging was also demonstrated [142]. In 2015, the N-719 derivative Ru(II) complex **31** was prepared by Fan and co-workers for colorimetric and luminescent determination of Hg^2+^ [144]. Similar to N-719, the response of complex **31** to Hg^2+^ was ascribed to the binding of electron-deficient Hg^2+^ to the electron-rich sulfur atom of NCS (thiocyante) groups. A 40-nm blue shift (from 525 to 485 nm) of absorption and a remarkable increase of luminescence at 720 nm were observed upon binding of complex **31** to Hg^2+^. In another study, Li et al. reported that a cyclometallated Ru(II) complex functionalized UCNPs for Hg^2+^ detection in water [145]. By modifying the cyclometallated Ru(II) complex with a propylsulfonate-coupled hemi-cyanine, Ru(II) complex **32** was then produced as the chemosensor for Hg^2+^ colorimetric analysis [146]. The Hg^2+^-initialized conversion of coordination from C to S atom led to a remarkable absorption and solution color change from dark red to light yellow. In a subsequent study, the same group modified sulfonate to carboxyl to prepare a polymer membrane for colorimetric detection of Hg^2+^ [147].

**Fig. 15:**
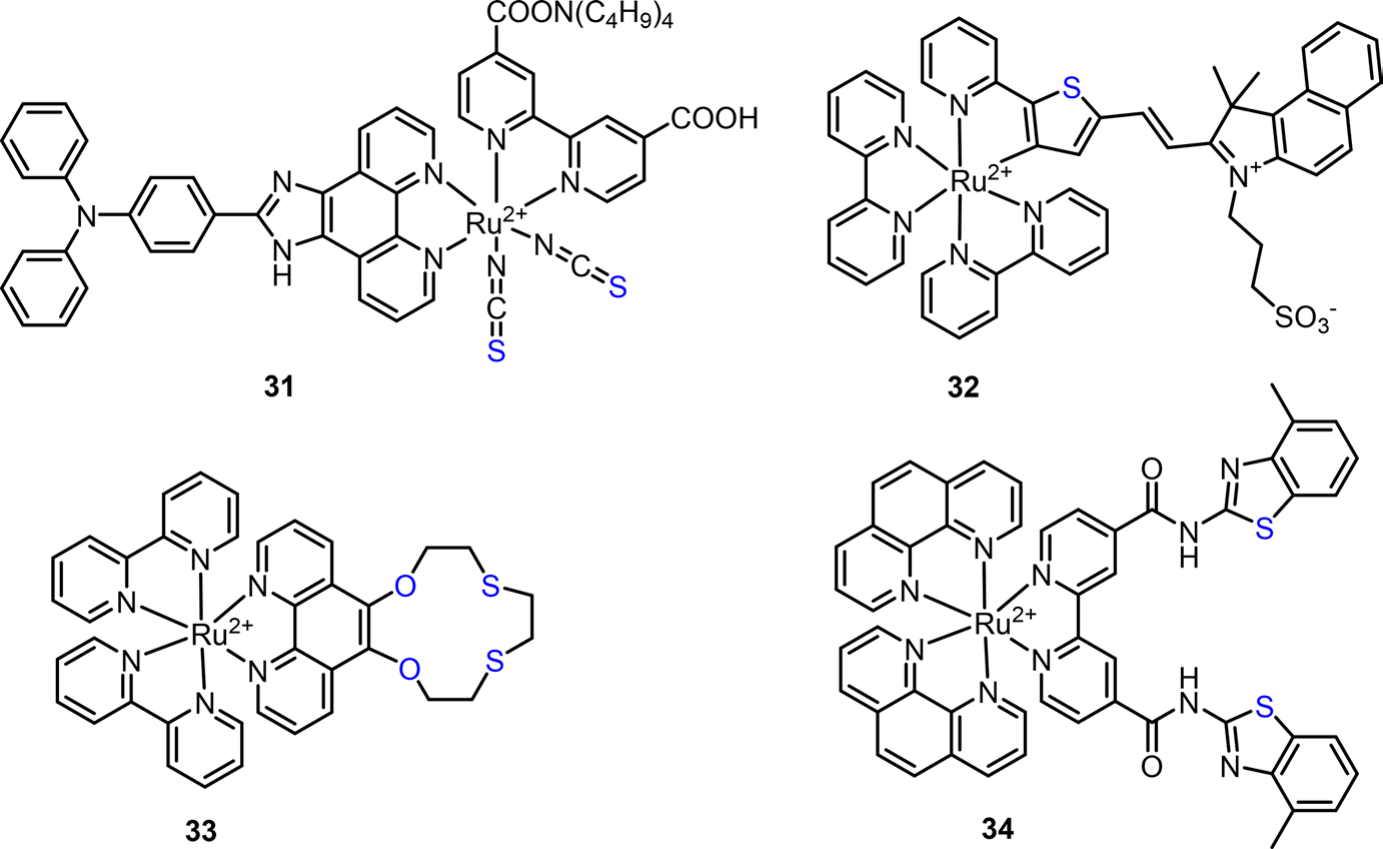
Molecular structure of Ru(II) complexes **31**–**34** for Hg^2+^ detection

The binding of S atom to Hg^2+^ has also been exploited for the development of Ru(II) complexes **33** and **34** for Hg^2+^ detection. For complex **33**, oxathiacrown ether is able to bind Hg^2+^, resulting in a 30 nm blue shift of absorption spectra and color change from red-orange to yellow [148]. For complex **34**, Hg^2+^ can bind to four benzothiazole S atoms from two complex **34**s through a 1:2 binding stoichiometry [149]. The response of Hg^2+^ was accompanied by a significant increase of complex **34**’s emission at 656 nm. Nevertheless, complex **34** showed poor selectivity to Hg^2+^, as luminescence enhancement at 630 nm was observed in the presence of Ag^+^. The response of complex **34** to Ag^+^ was due to the binding of Ag^+^ to benzothiazoles’ S atoms through a 1:1 stoichiometry (Fig. 15).

**Fig. 16:**
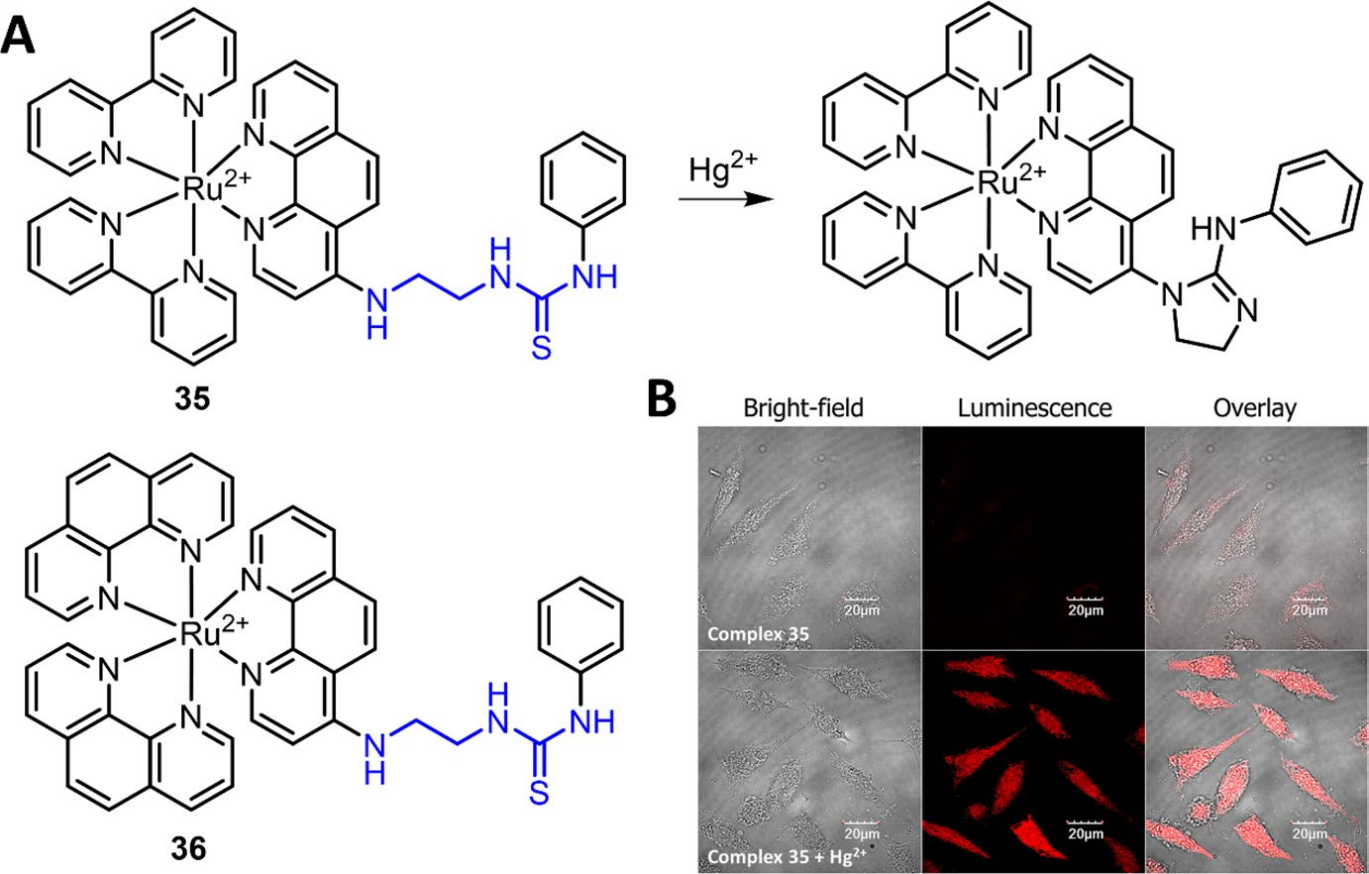
Molecular structure of Ru(II) complexes **35** and **36** and the reaction of complex **35** with Hg^2+^ (**A**). Luminescence imaging of Hg^2+^ in SMMC-7721 cells using complex **35** as the chemosensor (**B**). Adapted with permission from Ref. [150]. Copyright 2015 American Chemical Society

Based on the Hg^2+^-mediated desulfation and intramolecular cyclic guanylation of thiourea reaction, Ru et al. developed Ru(II) complex **35** as an “OFF–ON” luminescence response chemosensor for Hg^2+^ (Fig. 16A) [150]. Complex **35** showed weak luminescence (ϕ = 0.4%), while the emission significantly increased upon reacting with Hg^2+^ ϕ = 2.2%). This reaction-based chemosensor (**35**) showed high selectivity and sensitivity (LoD = 8 nM) for Hg^2+^ detection. More importantly, the long lifetime emission of Ru(II) complex (τ_35_ = 215 ns, τ_35-Hg_^2+^  = 785 ns) enabled the background-free TGL analysis of Hg^2+^. With this Ru(II) complex **35**, luminescence imaging of Hg^2+^ in SMMC-7721 cells was then demonstrated (Fig. 16B). Using the same response reaction, the same research group then developed a phen ligand-based Ru(II) complex **36** for Hg^2+^ detection (Fig. 16A) [151]. Compared with complex **35**, complex **36** has higher sensitivity with a detection limit down to 5.4 nM. Similarly, the applications of this chemosensor for TGL Hg^2+^ detection and luminescence imaging of Hg^2+^ in SMMC-7721 cells were also demonstrated.

### Ru(II) Complex Chemosensors for Other Metal Ions

**Fig. 17:**
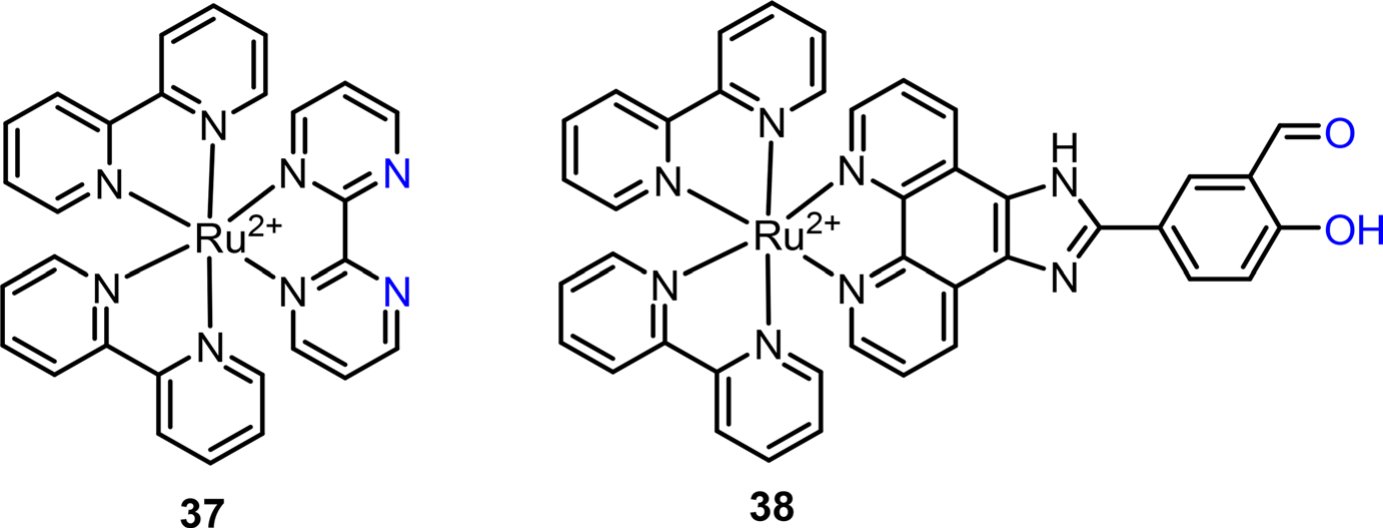
Molecular structures of Ru(II) complexes **37** and **38** for Pd^2+^ and Au^3+^ detection, respectively

Ru(II) complex-based chemosensors have also been developed for the detection of other metal ions [152], such as Fe^3+^ [153], Fe^2+^ [77], Na^+^ [154], Ag^+^ [149], Co^2+^ [155], Ba^2+^ [156] and Zn^2+^ [157]. For example, the 1,8-naphthyridine linked dinuclear Ru(II) complex has been reported for both Cu^2+^ and Fe^3+^ detection [153], and the azacrown ether coupled Ru(II) complex was found to respond to Ba^2+^ ion [156]. The high binding affinity of the tpy ligand with Fe^2+^ allowed Zheng et al. to use Ru(II) complex **18** for Fe^2+^ detection [158]. The coordination of Fe^2+^ with complex **18** through a 1:2 binding stoichiometry was accompanied by the quenching of Ru(II) complex’s emission at 608 nm. In 2014, Kumar and colleagues developed Ru(II) complex **37** for the detection of Pd^2+^ (Fig. 17) [159]. In the presence of Pd^2+^, a 16-nm blue shift of the MLCT absorption band and the emerging of a new absorption at 565 nm were observed, which was accompanied by a solution color change from orange to dark red. The emission at 670 nm of complex **37** (ϕ = 3.5%) was about 12-fold decreased (ϕ = 0.12%) because of the paramagnetic properties of Pd^2+^ ions. Recently, Xie and colleagues reported a Ru(II) complex **38** as the chemosensor for gold(III) (Au^3+^) detection (Fig. 17) [160]. Complex **38** showed high luminescence in PBS buffer (pH 7.2), while its emission was remarkably quenched after binding with Au^3+^. Complex **38** showed high sensitivity (LoD = 135 nM) and selectivity to Au^3+^, and low cytotoxicity, allowing its use for Au^3+^ imaging in living cells and zebrafish. The application of this chemosensor for Au^+^ drug release in anti-inflammation drugs was then demonstrated in inflamed microphage cells and LPS-induced zebrafish.

## Ru(II) Complex Chemosensors for Reactive Biomolecules

Ru(II) complexes have also been designed and synthesized as the chemosensors for the determination of small reactive biomolecules, including reactive nitrogen/oxygen/sulfur/carbonyl species (RNS/ROS/RSS/RCS) and amino acids. These biomolecules play very important roles in biological systems [25, 27, 161]. For example, ROS/RNS are important signaling molecules in the body, while high levels of ROS/RNS lead to oxidative stress, causing damage to the cell membrane, protein and nucleic acids [162]. It has been reported that the overexpression of ROS/RNS is implicated in pathological processes in inflammation, cancer, cardiovascular disease, Alzheimer’s disease (AD) and aging [162, 163]. Nevertheless, determination and monitoring their levels in situ remain a challenge due to (1) the limited numbers of robust chemosensors and (2) their high reactivity and the short lifetime of most species, particularly the ROS/RNS [99, 164–166]. This section will outline the contributions of Ru(II) complex chemosensors to the determination and imaging of these small biomolecules.

### Ru(II) Complex Chemosensors for RNS

**Fig. 18:**
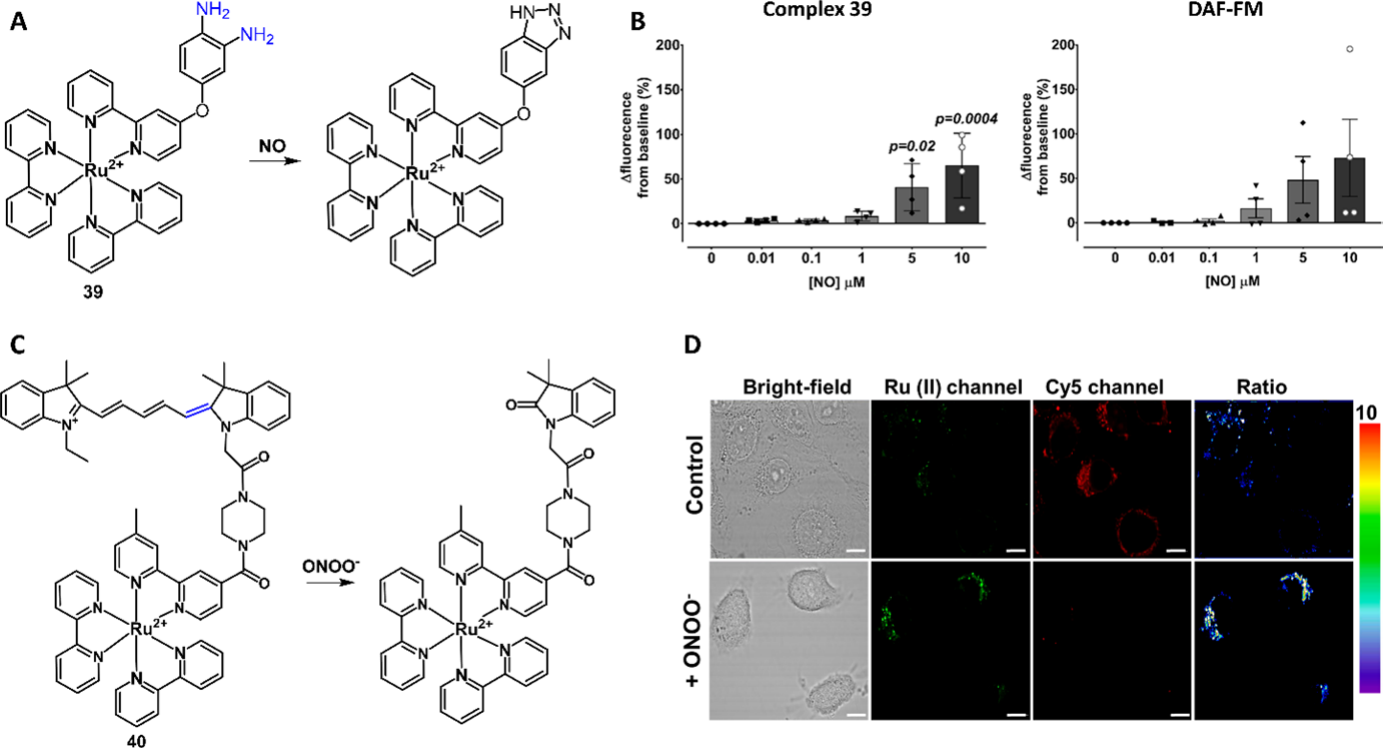
Molecular structure of Ru(II) complexes **39** and **40** and their response reaction with NO (**A**) and ONOO^−^ (**C**), respectively. Comparison of sensitivity of complex **39** with DAF-FM in cell-free media (**B**). Adapted with permission from Ref. [172]. Copyright 2019 Springer Nature. Luminescence ratiometric imaging of ONOO− in HeLa cells by complex 40 (D). Adapted with permission from Ref. [177]. Copyright 2018 Royal Society of Chemistry

RNS, mainly nitric oxide (NO), peroxynitrite (ONOO^−^) and nitroxyl (HNO), are endogenous biomolecules that play essential roles in various biological processes [167]. Of these RNS, NO is produced by conversion of arginine through nitric oxide synthase-mediated oxidation [167]. NO has been found to be associated with blood vessel health and signaling pathways [168]. The reaction of NO with superoxide (O_2_^•−^) forms ONOO^−^, a highly reactive biomolecule, contributing to signaling transduction in living systems [169]. In 2010, Zhang et al. pioneered the use of Ru(II) complexes for reactive biomolecule detection and demonstrated the first Ru(II) complex chemosensor for NO detection [170]. This Ru(II) complex (**39**) was simply designed by coupling of NO response unit, 3,4-diaminophenoxy, to Ru(II)-bpy luminophore (Fig. 18A). After reacting with NO, the electron density of the triazolephenoxy product was reduced, thus inhibiting the PeT process and turning “ON” the MLCT emission. Ru(II) complex **39** has high sensitivity and selectivity, and fast response to NO, allowing its further use for NO determination and imaging in animal and plant cells [170, 171]. Later research showed that complex **39** has better performance in endothelial NO detection compared with the DAF-FM (Fig. 18B), demonstrating the usefulness of complex **39** as a promising chemosensor for NO clinical investigations [172]. Further efforts have also been devoted to modifying complex **39** for the development of other chemosensors for NO detection [173–175].

To determine the levels of ONOO^−^ in living cells [176], Zhang et al. developed a Ru(II) complex **40** by coupling of Ru(II) complex with a cyanine 5 (Cy5) dyes (Fig. 18C) [177]. In this FRET-based chemosensor, the Ru(II) complex served as the energy donor, and Cy5 served as the energy acceptor. In the presence of ONOO^−^, the oxidation-cleavage led to the corruption of the electron transfer (ET) process [178], accompanied by the decrease of Cy5’s emission and increase of Ru(II) complex’s luminescence. Complex **40** also showed fast response, high sensitivity and selectivity to ONOO^−^, and low cytotoxicity, facilitating its application in ratiometric detection and imaging of mitochondrial ONOO^−^ in living HeLa cells (Fig. 18D).

### Ru(II) Complex Chemosensors for ROS

ROS is a group of reactive biomolecules and free radicals derived from molecular oxygen [162, 164, 179]. Generally, ROS include O_2_^•−^, hydroxyl radicals (•OH), hydrogen peroxide (H_2_O_2_), singlet oxygen (^1^O_2_) and hypochlorous acid (HOCl) [164]. These short-lived species display high reactivity, causing oxidation of other biomolecules, such as DNA, lipids and proteins [162]. In the past years, a number of Ru(II) complex chemosensors have been developed for the detection and imaging of ^1^O_2_ and HOCl [180], which will be discussed in this section.

**Fig. 19:**
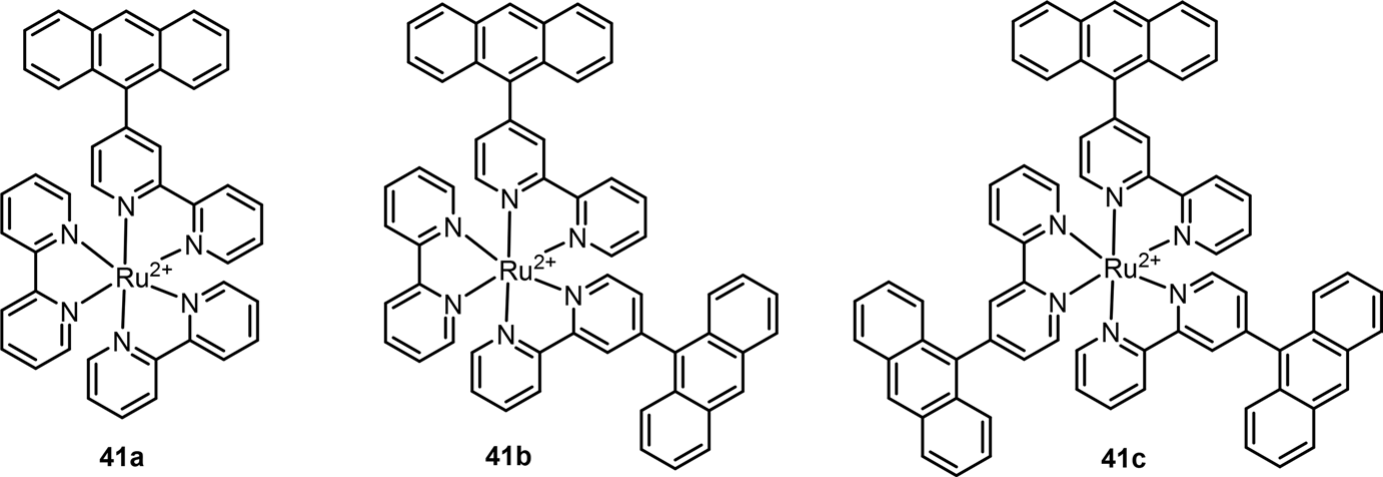
Molecular structures of Ru(II) complexes **41a**, **b**, **c** for ^1^O_2_ detection

Coupling with anthracene [181], pyrene [182] and 1,8-naphthalimide [183], early research revealed that Ru(II) complexes’ emissions can be quenched by the energy transfer mechanism. The triplet states of pyrene and 1,8-naphthalimide have similar energy levels to ^3^MLCT of Ru(II) complexes [46]; the energy transfer between these triplet states enables a longer emission lifetime with lower quantum yields compared with [Ru(bpy)_3_]^2+^ prototype complex [182–184]. Different from pyrene and 1,8-naphthalimide, the energy of anthracene’s triplet state lies 1460 cm^−1^ below the Ru(II) complexes’ ^3^MLCT [185], which leads to the quenching of ^3^MLCT states as the energy transfer from Ru(II) complexes’ ^3^MLCT to anthracene’s triplet state [181]. Upon specific oxidation of anthracene with ^1^O_2_, the corresponding endoperoxide is produced; thus, the quenching of ^3^MLCT is inhibited [186]. Inspired by this mechanism, Zhang et al. designed three Ru(II) complexes (**41a, b, c**) for ^1^O_2_ detection in buffer (Fig. 19) [187]. All three complexes showed high selectivity to ^1^O_2_ over other ROS and RNS, and the LoD was 0.17 μM, using **41a** as the chemosensor.

**Fig. 20:**
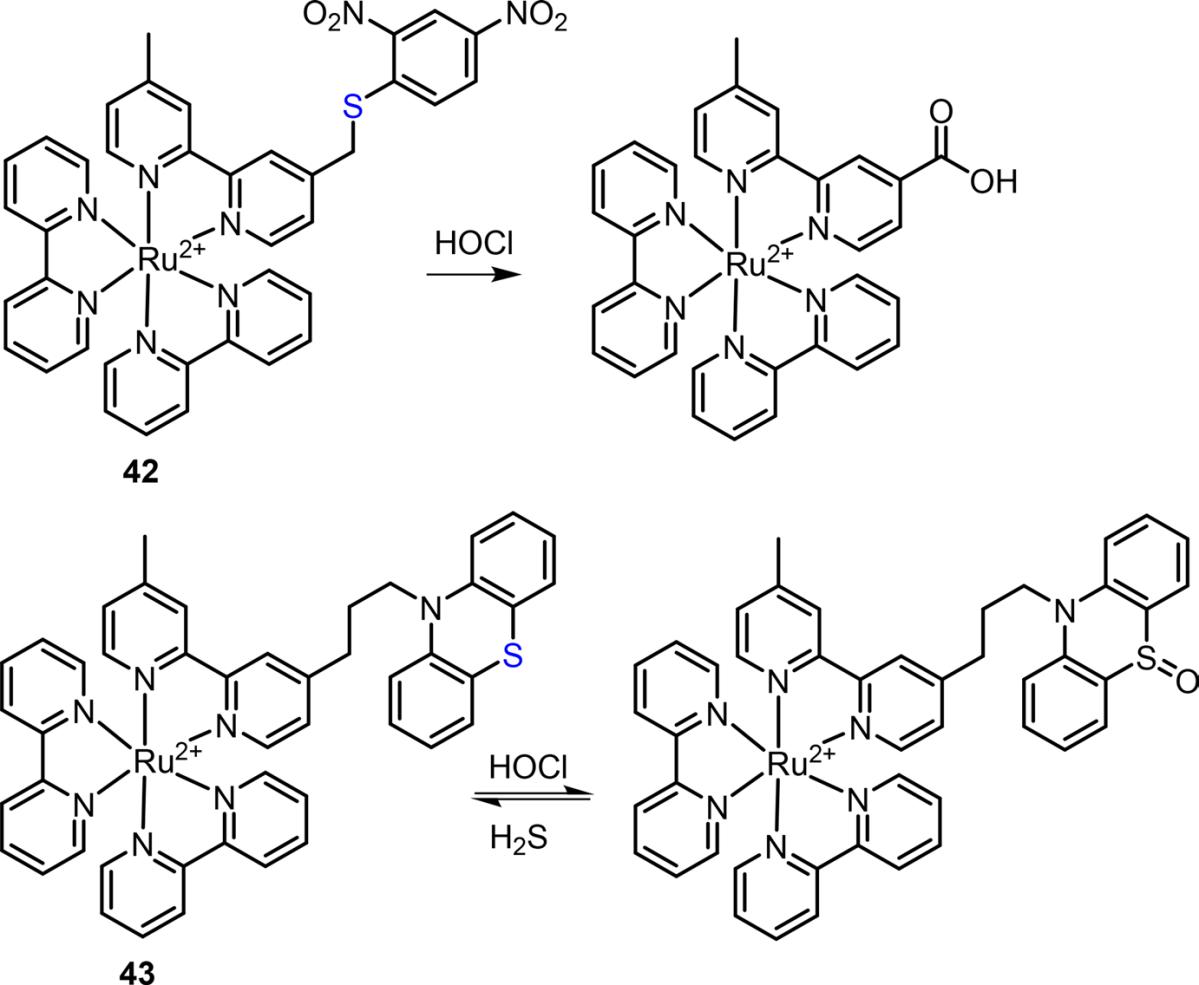
Molecular structure of Ru(II) complexes **42** and **43** and the oxidations with HOCl

For the determination of HOCl, Ru(II) complex chemosensors have been developed by exploiting different response reactions, including (1) oxidation of S atom [188, 189], (2) amines (including dibenzoylhydrazine) [190, 191] and (3) oxime derivatives and others [192, 193]. In 2013, Zhang et al. reported a Ru(II) complex chemosensor **42** for HOCl detection and imaging [188]. Complex **42** was developed by conjugating a Ru(II) complex luminophore and 2,4-dinitrophenyl (DNP) quencher through an S linker (Fig. 20). The specific oxidation of the S linker with HOCl led to the cleavage of electron transfer acceptor (DNP quencher), accompanied by an increase of MLCT emission at 626 nm. The applications of complex **42** for imaging of exogenous HOCl in HeLa and endogenous HOCl in RAW 264.7 cells were then demonstrated. HOCl-mediated oxidation of phenothiazine’s “S” has also been exploited for developing Ru(II) complexes for HOCl detection in biological [194] and environmental samples [189]. For example, in 2014, Liu et al. reported a Ru(II) complex **43** as the reversible chemosensor for HOCl determination and imaging (Fig. 20) [194]. An early study found that the Ru(II) complex emissions can be quenched by phenothiazine through a PeT mechanism, and the oxidation of “S” by Cu^2+^ resulted in the “OFF–ON” luminescent response of complex **29** for Cu^2+^ detection [135]. The oxidation of phenothiazine’s “S” can also be triggered by HOCl, enabling complex **43** to be used for HOCl detection by recording the emission changes at 605 nm. More interestingly, the sulfoxide oxidation product can be reduced by H_2_S, resulting in a reversible chemosensor for HOCl detection.

**Fig. 21:**
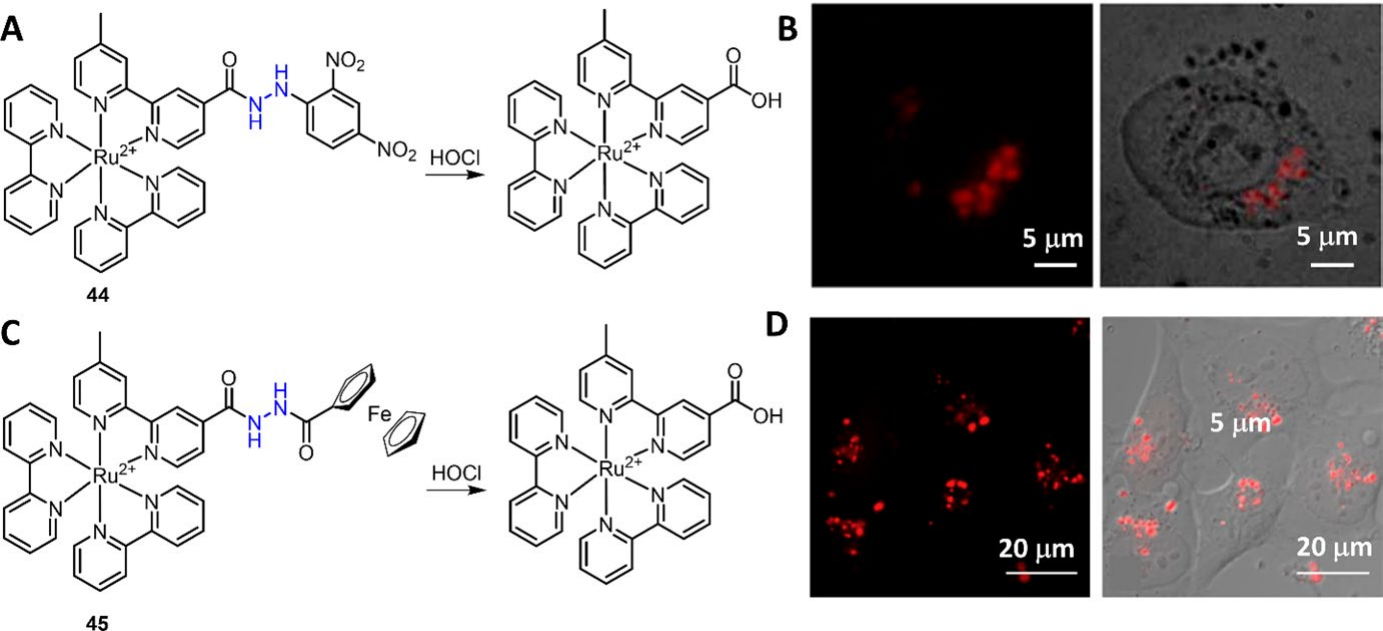
Molecular structure of Ru(II) complexes **44** (**A**) and **45** (**C**) and oxidations with HOCl. Luminescence imaging of phagocytosis-induced HOCl production in RAW 264.7 macrophage cells using Ru(II) complex **44** (**B**) and lysosomal HOCl in MDA-MB-231 cells using Ru(II) complex **45** (**D**). Adapted with permission from Ref. [191, 195]. Copyright 2013, 2015 Elsevier

In 2013, HOCl-mediated oxidation of amide was exploited by Zhang et al. for the development of Ru(II) complex chemosensor **44** for HOCl detection (Fig. 21) [195]. The emission of Ru(II) complex luminophore was quenched by the PeT from Ru(II) center to DNP quencher. In the presence of HOCl, fast oxidation-cleavage of DNP allowed the production of the luminescent Ru(II) complex. More than 1100-fold enhancement in luminescence at 626 nm was obtained immediately after the reaction of complex **44** with HOCl. The application of complex **44** for imaging of phagocytosis-induced HOCl production in RAW 264.7 macrophage cells was then successfully demonstrated. The decrease and blue shift of an absorption band at about 450 nm allowed further development of complex **44**-loaded UCNPs for background-free luminescent detection of HOCl [53]. In a subsequent study, Zhang et al. reported Ru(II) complex **45** for HOCl detection and lysosomal imaging (Fig. 21) [191]. Complex **45** was developed by coupling of the Ru(II) complex and ferrocenyl moiety through a HOCl-responsive hydrazine linker. Complex **45** can be internalized through a caveolae-mediated endocytosis process. The lysosomal accumulation of complex **45** allowed its use for imaging of lysosomal HOCl. The application of complex **45** in imaging of HOCl in vivo, including in flea and zebrafish, was then demonstrated.

**Fig. 22:**
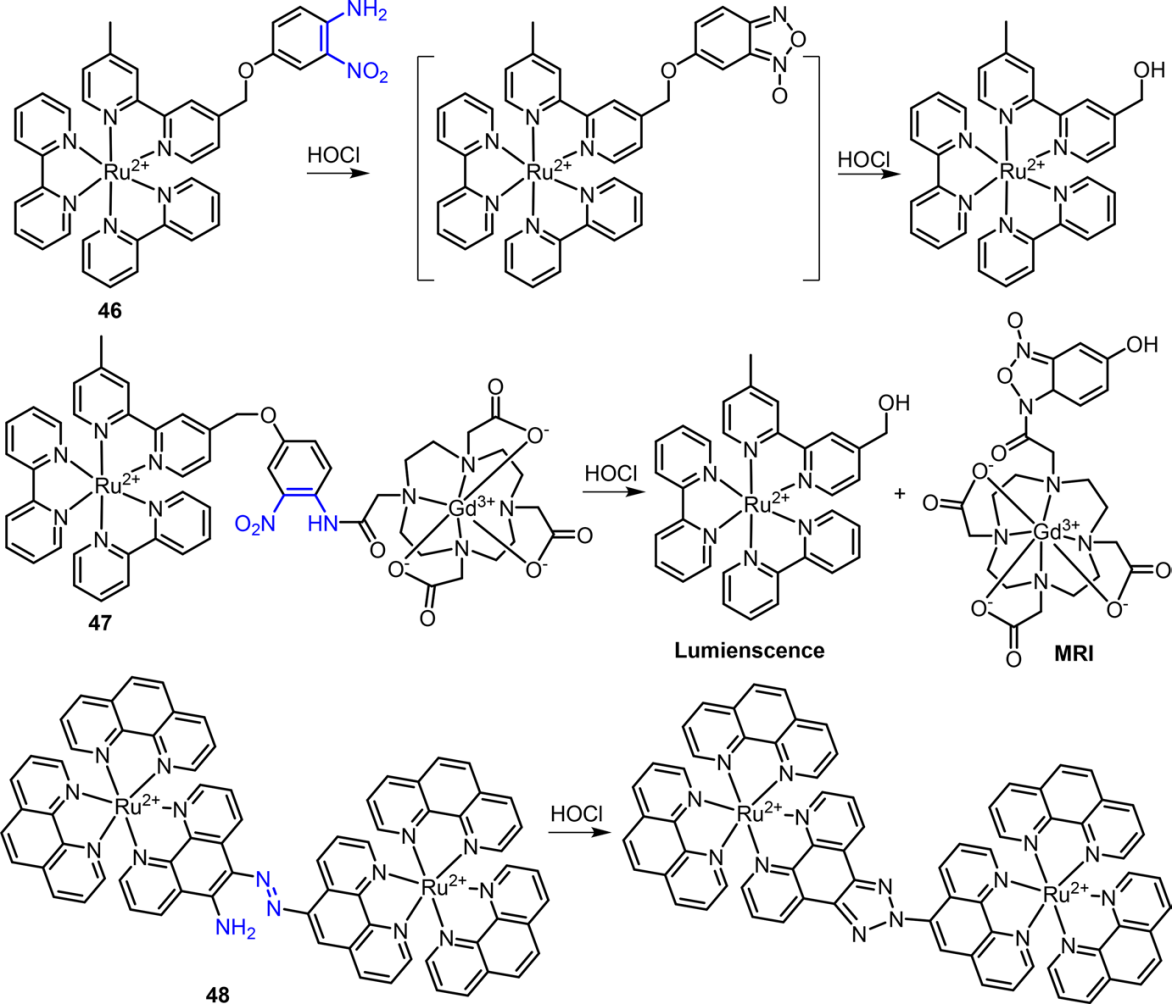
Molecular structure of Ru(II) complexes **46**–**48** and their oxidations with HOCl

*O*-nitroaniline can react specifically with HOCl to form a benzofurazan-1-oxide (BFO) in aqueous solution. Based on this reaction, Zhang et al. developed Ru(II) complex **46** as a chemosensor for HOCl detection (Fig. 22) [196]. The MLCT emission was quenched because of the PeT mechanism, while the products of complex **46** reacting with HOCl showed intense emission in borate buffer. Complex **46** was then applied to imaging of exogenous HOCl in HeLa and endogenous HOCl in neutrophils. In a subsequent study, Shi et al. modified complex **46** with a Gd-DOTA contrast agent to produce the hetrobimetallic Ru(II)–Gd(III) complex **47** for bimodal (luminescence and magnetic resonance imaging (MRI)) determination and imaging of HOCl (Fig. 22) [197]. Upon reacting with HOCl, both luminescence and MR signals were increased. The increase of luminescence was attributed to the corruption of PeT between *o*-nitroaniline and Ru(II) complex, and the increase of MR signal was ascribed to the increased number of inner-water molecules. Then, complex **47** was applied to luminescence and MRI detection of HOCl in drug-induced acute liver and kidney injury in a mouse. Following the development of complex **30** for Cu^2+^ detection [136], the same group found that the dinuclear Ru(II) complex **48** can also be used for HOCl detection (Fig. 22) [198]. The oxidation-cyclization of the azo and amino group in complex **48** produced a triazole derivative, accompanied by > 50-fold enhancement of MLCT emission at 600 nm. Moreover, this oxidation-cyclization showed high selectivity to HOCl over other ROS and Cu^2+^. Complex **48** was then applied to luminescence imaging of HOCl in mouse.

### Ru(II) Complex Chemosensors for RCS

RCS is a family of small and transient carbon-based metabolites that are implicated in several biological process [199]. These species can react with proteins through covalent bonds. As a result, the functions of proteins are changed, and thus the biological processes of these proteins are affected. In living organisms, the RCS mainly include glyoxal (GO), carbon monoxide (CO), formaldehyde (FA) and methylglyoxal (MGO). Although Ru(II) complexes have been widely investigated in developing chemosensors for ions and ROS/RNS, only a few complexes have been reported for RCS detection and imaging, which will be outlined in this section.

**Fig. 23:**
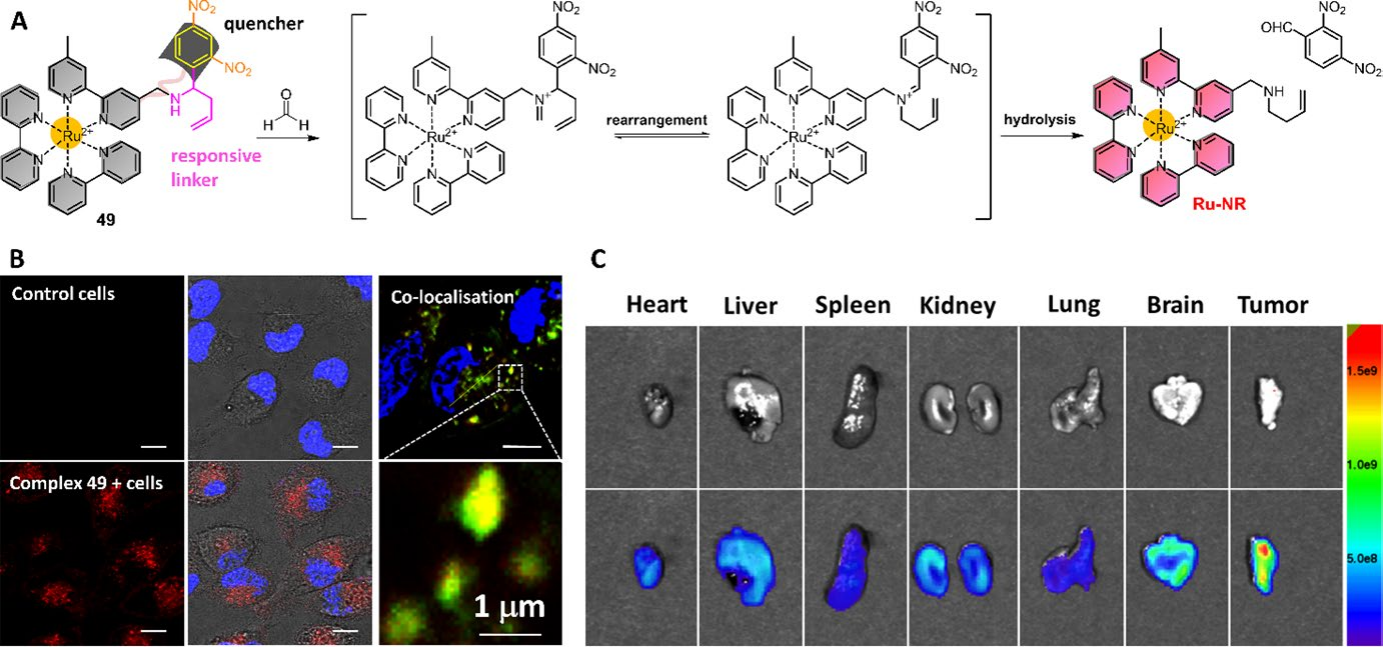
Molecular structure of Ru(II) complex **49** and its response reaction with FA (**A**). The luminescence imaging of intracellular FA in lysosomes (**B**) and ex vivo imaging of FA in different mouse organs (**C**). Adapted with permission from Ref. [202]. Copyright 2019 American Chemical Society

Ru(II) complex with 4,5-diamino-1,10-phenanthroline ligand has been previously employed as the chemosensor for NO detection [173]. Recent research found that the Ru(II) complexes’ 4,5-diamino-1,10-phenanthroline ligand can respond to RCS [200], particularly the MGO to form 2-methylpyrazino-1,10-phenanthroline ligand coordinated products [201]. Based on this reaction, Zhang et al. investigated the capability of the Ru(II) complex chemosensor for MGO determination and imaging in RAW 264.7 macrophages and flea. Recently, Zhang et al. reported a “dual-key-and-lock” Ru(II) complex chemosensor **49** for lysosomal FA determination in cancer cells and tumors (Fig. 23A) [202]. Complex **49** was designed by coupling of Ru(II) complex with a DNP quencher through an FA-responsive linker. Interestingly, the response reaction of complex **49** can only take place in the presence of FA (first “key”) under acidic conditions (second “key”), which allow FA detection specifically in lysosomes. Complex **49** has a long lifetime (τ = 330.4 ns), which facilitates the application of background-free TGL analysis in human serum samples and mouse organs. Luminescence imaging results clearly showed that complex **49** could be used for lysosomal FA detection in HeLa cells (Fig. 23B). With this FA-responsive Ru(II) complex, in vivo and ex vivo imaging results confirmed the much higher FA levels in tumor cells and tissues (Fig. 23C).

### Ru(II) Complex Chemosensors for RSS

Hydrogen sulfide (H_2_S) is one of the major RSS in the human body and is involved in various biological processes. This endogenous gaseous molecule is produced by CBS (cystathionine β-synthase) and CSE (cystathionine γ-lyase) catalyzed reaction with thiol-containing biomolecules [203]. Recent research has also revealed that the H_2_S is a gasotransmitter and a regulator of critical biological processes [204, 205]. The metabolites of H_2_S, such as polysulfides and persulfides, are also important RSS that may have similar or divergent regulatory roles in living systems [206, 207]. Recently, by exploiting the response mechanisms of sulfoxide reduction [194], displacement of Cu^2+^ [137], cleavage of 7-nitro-2,1,3-benzoxadiazoles (NBD) [208] and DNP [209], a few Ru(II) complexes have been developed as chemosensors for RSS detection [194], and these RSS-responsive Ru(II) complexes will be discussed in this section.

**Fig. 24:**
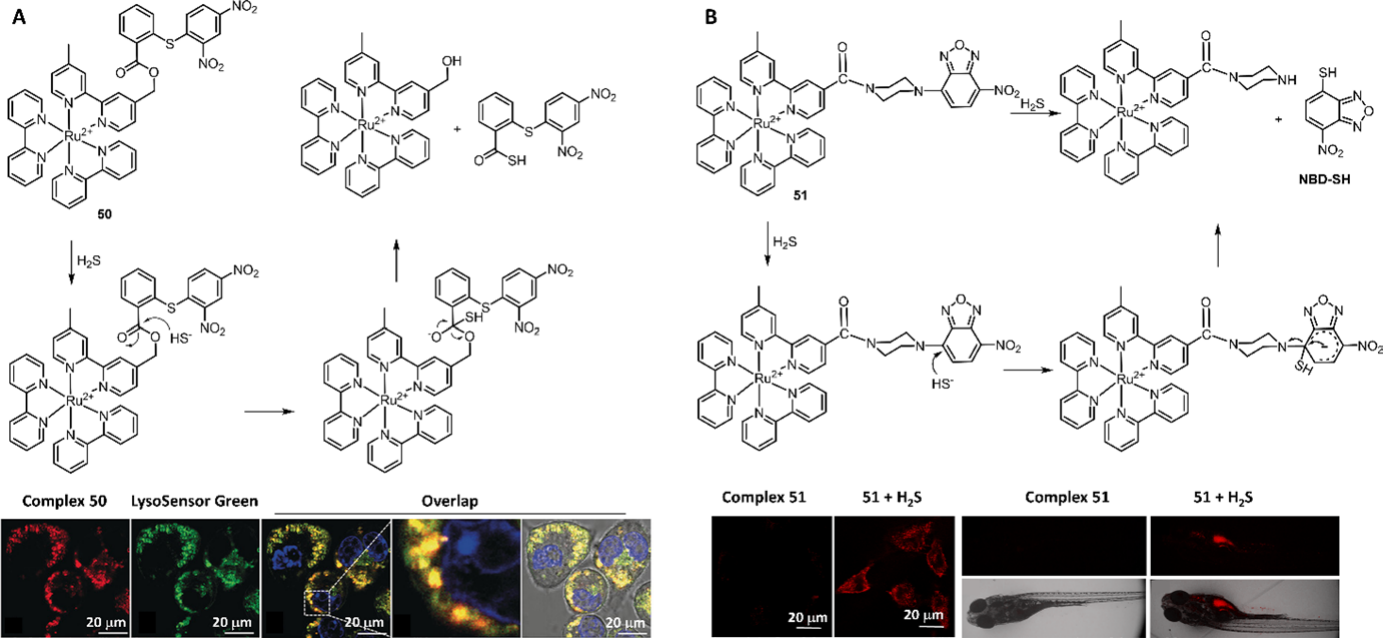
Molecular structures of complex **50** (**A**) and **51** (**B**) and the application of complex **50**, **51** for H_2_S imaging in cells and zebrafish. Adapted with permission from Ref. [208, 209]. Copyright 2018 Wiley and 2021 Elsevier

In 2018, coupling of Ru(II) luminophore and DNP quencher with a H_2_S-responsive linker, Du et al. reported Ru(II) complex **50** as the chemosensor for H_2_S (Fig. 24A) [209]. Complex **50** was weakly luminescent because of the PeT from the Ru(II) center to DNP. In the presence of H_2_S, the cleavage of the linker led to more than 86-fold enhancement in emission intensity at 612 nm. The long lifetime (> 300 ns) of both complex **50** and the products facilitated the TGL assay of H_2_S in human blood serum samples and mouse organs without any background signals. The H_2_S concentration was 47.70 ± 4.50 μM and < 1.2 μmol/g in sera and mouse organs, respectively. Luminescent imaging results showed that complex **50** accumulated in lysosomes, thus allowing lysosomal H_2_S to be detected. By exploiting the H_2_S-triggered thiolysis of NBD, Ru(II) complex **51** was recently developed by Liu et al. for the detection of H_2_S in PBS buffer and in living organisms (Fig. 24B) [208]. Similar to complex **50**, the emission quenching of complex **51** was attributed to the PeT from the Ru(II) center to DNB. The thiolysis of complex **51** showed high sensitivity (LoD = 177.3 nM) and selectivity to H_2_S over other biothiols, including Cys, Hcy and glutathione (GSH). Complex **51** was then applied to visualize the H_2_S in living HeLa cells and zebrafish.

## Ru(II) Complex Chemosensors for Amino Acids

Amino acids are building blocks of proteins and play critical roles in living organisms [210]. In living organisms, the amino acids are involved in synthesis of proteins and other nitrogen-containing species, such as hormones, enzymes, creatine and some neurotransmitters. In the past few years, luminescent Ru(II) complexes have contributed significantly to the development of chemosensors for various amino acids, such as biothiols (Cys, Hcy, and GSH), histidine (His) and others, which will be discussed in this section.

### Ru(II) Complex Chemosensors for Biothiols

GSH is one of the most abundant biothiols in the human body with intracellular concentrations ranging from 1 to 10 mM and about 1 mM in blood [161]. The intracellular Cys concentration is in the range of 30–200 μM and about 250 μM in blood [161]. Different from GSH and Cys, Hcy has a much lower concentration in the body, i.e., 5–15 μM in cells and about 10 μM in blood [211]. Recent studies have also revealed that the concentration of biothiols is implicated in different conditions, such as inflammation, cardiovascular diseases, HIV infection and cancers [161]. To detect biothiols in the body, a series of Ru(II) complex chemosensors have been developed through the following response mechanisms, (1) nucleophilic substitution and cleavage of the sulfonamide or sulfonate ester bond [212–214], (2) cyclization of aldehyde group with amino and thiol groups [100, 215, 216] and (3) others [217], such as reaction with azo group [218] and α,β-unsaturated ketone [219], cleavage of NBD and displacement of metal ions [139, 220].

**Fig. 25:**
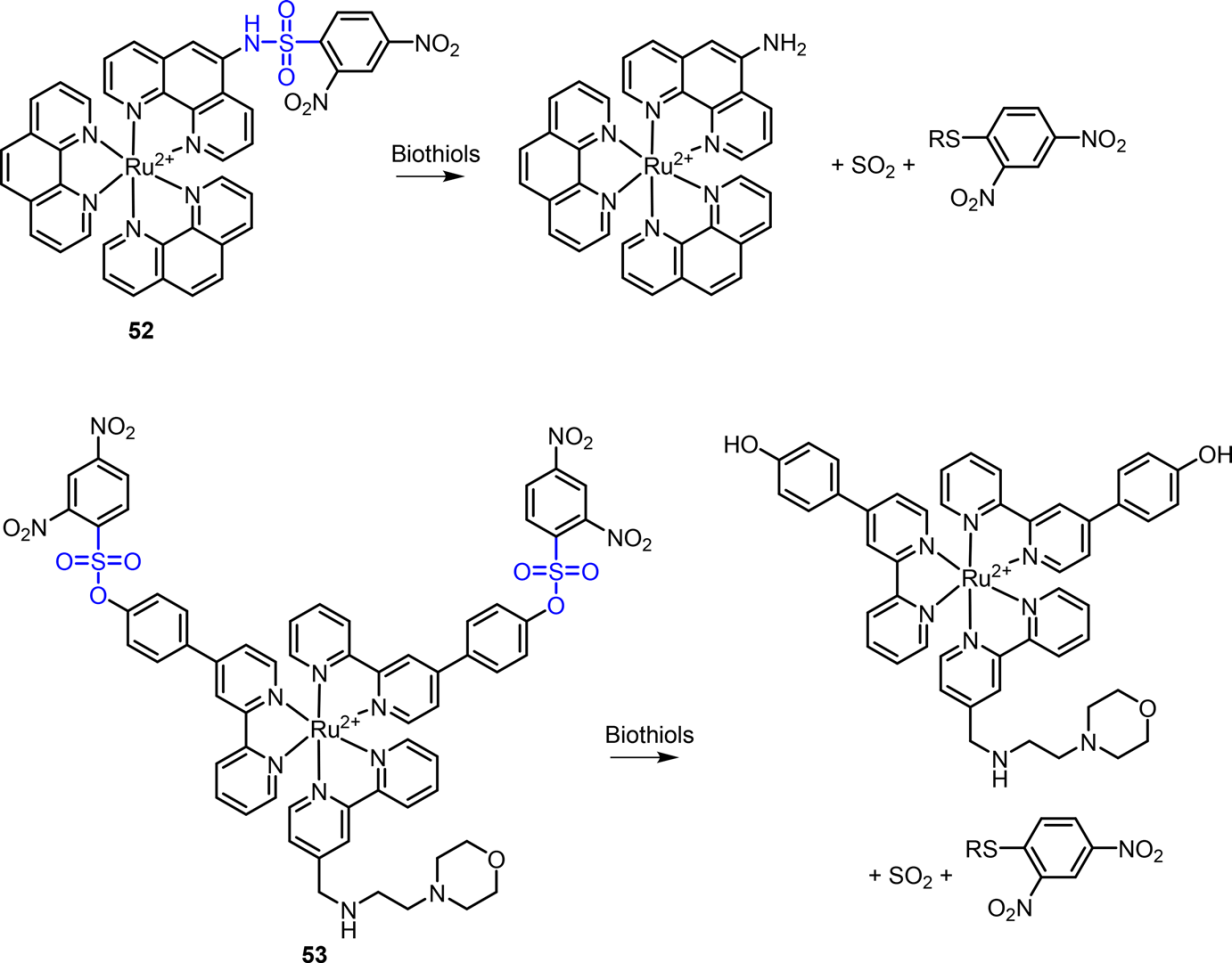
Molecular structures of Ru(II) complex **52** and **53** and their response reactions with biothiols

Through coupling of 2,4-dinitrobenzenesulfonyl to the amine of phen ligand, Ru(II) complex **52** was developed for biothiols determination by Ji and co-workers in 2010 (Fig. 25) [221]. Upon the reaction with biothiols, cleavage of the DNP electron acceptor led to a 90-fold enhancement of MLCT emission at 598 nm. Imaging of biothiols in NCI-H446 cells was then demonstrated using complex **52** as chemosensor. In a prior study, Zhang et al. found that the Ru(II) complexes with two response units could have higher “OFF-to-ON” ratios for thiophenol detection [222]. In 2017, Gao et al. reported on Ru(II) complex **53** for luminescence detection of biothiols (Fig. 25) [223]. In this Ru(II) complex, two DNP quenchers were linked to two bpy ligands through a sulfonate ester bond, which enabled the quenching of MLCT emission and the “OFF–ON” response to biothiols. A morpholine moiety was conjugated to the third bpy ligand, allowing complex **53** with lysosome targeting ability. The capability of complex **53** for background-free TGL detection of biothiols was also demonstrated.

**Fig. 26:**
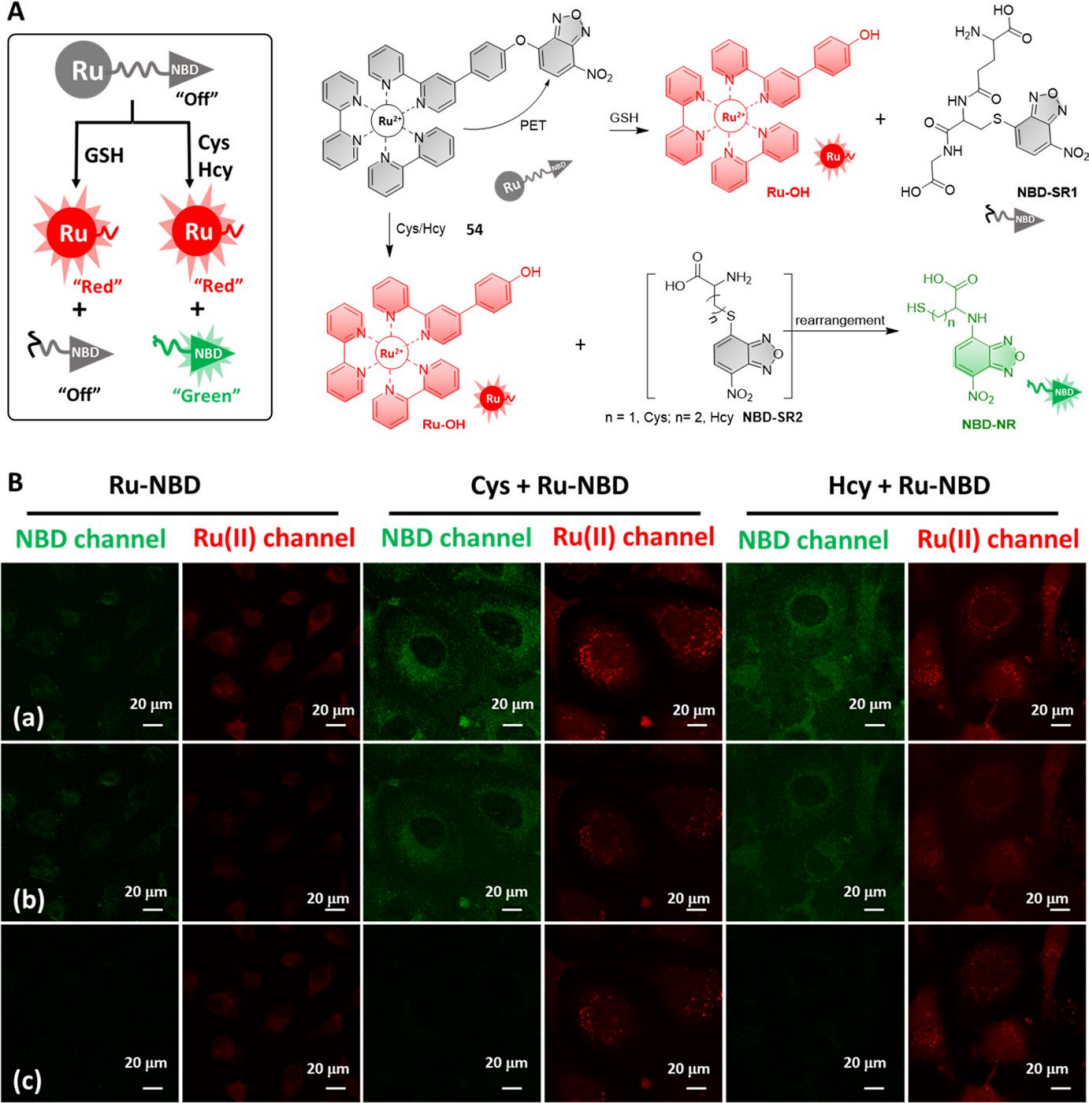
Strategy for the development of Ru(II) complex **54** for biothiol detection and discrimination (**A**). Luminescence and TGL imaging (a, 0–12 ns; b, 0–4 ns; c, 4–12 ns) of HeLa cells incubated with complex **54** (**B**). Adapted with permission from Ref. [224]. Copyright 2020 Wiley

Although a number of biothiol-sensitive chemosensors have been reported, the measurement of total biothiols and determination the level of each one remains a challenge. In 2020, Liu et al. reported a “Two Birds with One Stone” Ru(II) complex **54** for the detection and discrimination of biothiols in vitro and in vivo (Fig. 26A) [224]. Complex **54** was developed through coupling of two different signaling units (Ru(II) complex and NBD) through a “luminophore-responsive linker-luminophore” approach. In the presence of GSH, the cleavage of “O” ether bond led to the formation of a luminescent Ru(II) complex and non-fluorescent NBD-SR1. In contrast, the reaction of complex **54** with Cys and Hcy led to the formation of a red-emitting Ru(II) complex and NBD-SR2 that can further undergo a five- or six-member cyclic intermediate-associated rearrangement to form corresponding green-emitting NBD-NR. This allowed for discrimination of GSH from Cys and Hcy under steady-state luminescence measurements. Moreover, under the TGL measurement model, the total biothiol concentration was obtained as elimination of the emission from NBD-NR. The GSH and Cys/Hcy concentrations were thus determined by measuring the same sample with both steady-state and TGL models. The time-gated luminescence imaging of intracellular biothiols was then demonstrated, showing that the NBD emission was eliminated after a 4-ns delay (τ_NBD-NR_ = 0.8 ns) (Fig. 26B).

### Ru(II) Complex Chemosensors for Other Amino Acids

**Fig. 27:**
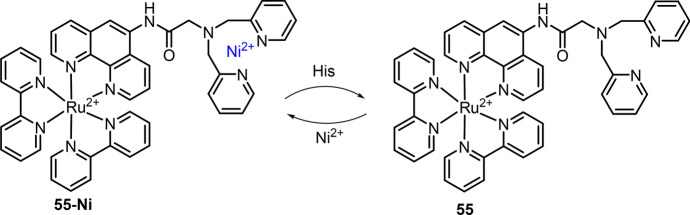
Molecular structure of Ru(II) complex **55** and heterobimetallic Ru(II)–Ni(II) complex for His detection

Ru(II) complexes have also been developed for the detection of other amino acids, such as methionine (Met) and histidine (His), through the response mechanism of amino acid-dominated binding of metal ions (e.g., Cu^2+^ and Ni^2+^) [140, 220, 225]. For example, Gao et al. developed Ru(II) complex **55** in 2015 and then used the heterobimetallic Ru(II)–Ni(II) complex as the chemosensor for His detection (Fig. 27) [226]. Complex **55** showed intense luminescence in EtOH/HEPES buffer (50 mM, pH 7.2, 2:3, v/v), and its emission was quenched upon binding to Ni^2+^. In the presence of His, the displacement of Ni^2+^ led to the recovery of Ru(II) complex’s emission at 603 nm. The LoD was 265 nM at this test condition. Imaging of His in HeLa cells, zebrafish and flea was then performed using complex **55** as the chemosensor.

## Conclusions

The past few decades have witnessed rapid progress in the development of chemosensors for biological investigations and environmental sample determinations. Of various chemosensors, the ones using Ru(II) complex-based luminophores are particularly interesting because of their abundant photo-physical/-chemical properties. Consequently, a series of Ru(II) complex chemosensors have been developed for the detection of ions and small biomolecules in recent years. In this review, the progress in the development of Ru(II) complex chemosensors for the detection of anions, metal ions, reactive biomolecules (ROS, RNS, RSS, and RCS) and amino acids was summarized, particularly focused on those chemosensors that can be used for target analyte detection in aqueous solution and imaging in living systems.

By carefully surveying the reported Ru(II) complex chemosensors, it is clear that the Ru(II) complex has been a useful platform for the determination of various analyte levels in vitro and in vivo. Nevertheless, there is still some room for further development of Ru(II) complex chemosensors in future studies. As it has been confirmed that the photo-physical/-chemical properties of Ru(II) complexes are varied by changes of the coordination ligands [227–230], the toxicity, particularly the photo-toxicity, could be one of the key considerations in developing Ru(II) complex chemosensors in the future. Generally, low cytotoxicity is one of the essential criteria for a chemosensor to be applied in biological studies. Moreover, the triplet nature of the emission state of Ru(II) complexes with long phosphorescence lifetime is easily quenched by oxygen [56]. Therefore, development of the Ru(II) complex chemosensors with minimal quenching from the surrounding environments, particularly the levels of oxygen, is also demanded. With intense background autofluorescence in biological systems limiting the use of other probes, recent research has confirmed that indeed Ru(II) complexes with prolonged emission lifetime can be used for TGL bioassays and imaging [28, 31, 202]. Such a background-free bioassay and imaging approach allow the determination of target analytes in living cells with higher sensitivity and signal-to-noise (S/N) ratio, which can be further investigated for biomolecule detection in vivo in future studies.

In summary, taking together with the unique photo-physical/-chemical properties of Ru(II) complexes and the potential applications of chemosensors, ongoing research is expected to develop robust Ru(II) complex chemosensors for the determination and imaging of ions and biomolecules in the future. We hope that this review will provide a knowledge base for the Ru(II) complex chemosensor area and inspire the readers to contribute to this promising research field in the future.

## Abbreviations

Ru(II): Ruthenium(II)
HPLC: High-performance liquid chromatography
ICP-OES/MS: Inductively coupled plasma-optical emission spectroscopy/mass spectrometry
bpy: 2,2′-Bipyridine
CT: Computerized tomography
MRI: Magnetic resonance imaging
PET: Positron emission tomography
CA: Contrast agent
LoD: Limit of detection
ϕ: Quantum yields
TGL: Time-gated luminescence
Ir(III): Iridium(III)
Pt(II): Platinum(II)
Au(I): Gold(I)
Re(I): Rhenium(I)
Os(II): Osmium(II)
MLCT: Metal-to-ligand charge transfer
ILCT: Intraligand charge transfer
LLCT: Ligand-to-ligand charge transfer
MMLCT: Metal–metal-to-ligand charge transfer
LMCT: Ligand-to-metal charge transfer
MLLCT: Metal-to-ligand-ligand charge transfer
LMMCT: Ligand-to-metal–metal charge transfer
MC: Metal centered
LC: Ligand centered
FRET: Förster resonance energy transfer
PeT: Photo-induced electron transfer
dppz: Dipyridophenazine
ROS: Reactive oxygen species
RNS: Reactive nitrogen species
RCS: Reactive carbonyl species
RSS: Reactive sulfur species
F^−^: Fluoride
CH_3_COO^−^: Acetate
CN^−^: Cyanide
H_2_PO_4_^−^: Phosphate
Cl^−^: Chloride
Br^−^: Bromide
CH_3_CN: Acetonitrile
DMSO: Dimethyl sulfoxide
SCN^−^: Thiocyanate
Cys: Cysteine
Hcy: Homocysteine
GSH: Glutathione
HSO_3_^−^: Hydrogen sulfite
S^2^^−^: Sulfide
PPi: Pyrophosphate
DPA: Di(2-picolyl)amine
ATP: Adenosine triphosphate
UCNP: Upconversion nanoparticle
UCL: Upconversion luminescence
NO: Nitric oxide
ONOO^−^: Peroxynitrite
HNO: Nitroxyl
O_2_^•^^−^: Superoxide
Cy5: Cyanine 5
•OH: Hydroxyl radicals
H_2_O_2_: Hydrogen peroxide
^1^O_2_: Singlet oxygen
HOCl: Hypochlorous acid
DNP: 2,4-Dinitrophenyl
BFO: Benzofurazan-1-oxide
GO: Glyoxal
CO: Carbon monoxide
MGO: Methylglyoxal
FA: Formaldehyde
CBS: Cystathionine β-synthase
CSE: Cystathionine γ-lyase
H_2_S: Hydrogen sulfide
NBD: 7-Nitro-2,1,3-benzoxadiazoles
His: Histidine
S/N: Signal-to-noise
